# Establishment of a circRNA-regulated E3 ubiquitin ligase signature and nomogram to predict immunotherapeutic efficacy and prognosis in hepatocellular carcinoma

**DOI:** 10.1186/s40001-024-01893-6

**Published:** 2024-06-10

**Authors:** Gefeng Wu, Jiahao Zhang, Rui Peng, Jun Cao, Daoyuan Tu, Jie Zhou, Bingbing Su, Shengjie Jin, Guoqing Jiang, Chi Zhang, Dousheng Bai

**Affiliations:** 1 Department of Hepatobiliary Surgery, Clinical Medical College, Yangzhou University, 98 West Nantong Rd, Yangzhou, 225000 Jiangsu China; 2 Dalian Medical University, Dalian, 116000 China

**Keywords:** Hepatocellular carcinoma, CircRNA, E3 ubiquitin ligase signature, Nomogram, Immune and prognosis

## Abstract

**Background:**

Hepatocellular carcinoma (HCC) is a common type of malignant tumor where the prognosis is dismal. Circular RNA (CircRNA) is a novel RNA that regulates downstream gene transcription and translation to influence the progression of HCC. However, the regulatory relationship that exists between E3 ligases, which is a class of post-translational modifying proteins, and circRNA remains unclear.

**Methods:**

Based on the E3 ubiquitin ligase in the competitive endogenous RNA (ceRNA) network, a circRNA-regulated E3 ubiquitin ligase signature (CRE3UL) was developed. A CRE3UL signature was created using the least absolute shrinkage and selection operator (Lasso) and Cox regression analysis and merged it with clinicopathologic characteristics to generate a nomogram for prognosis prediction. The pRRophetic algorithm was utilized and immunological checkpoints were analyzed to compare the responses of patients in the high-risk group (HRG) and low-risk group (LRG) to targeted therapy and immunotherapy. Finally, experimental research will further elucidate the relationship between E3 ubiquitin ligase signature and HCC.

**Results:**

HRG patients were found to have a worse prognosis than LRG patients. Furthermore, significant variations in prognosis were observed among different subgroups based on various clinical characteristics. The CRE3UL signature was identified as being an independent prognostic indicator. The nomogram that combined clinical characteristics and the CRE3UL signature was found to accurately predict the prognosis of HCC patients and demonstrated greater clinical utility than the current TNM staging approach. According to anticancer medication sensitivity predictions, the tumors of HRG patients were more responsive to gefitinib and nilotinib. From immune-checkpoint markers analysis, immunotherapy was identified as being more probable to assist those in the HRG.

**Conclusions:**

We found a significant correlation between the CRE3UL signature and the tumor microenvironment, enabling precise prognosis prediction for HCC patients. Additionally, a nomogram was developed that performs well in predicting the overall survival (OS) of HCC patients. This provides valuable guidance for clinicians in devising specific personalized treatment strategies.

**Graphical Abstract:**

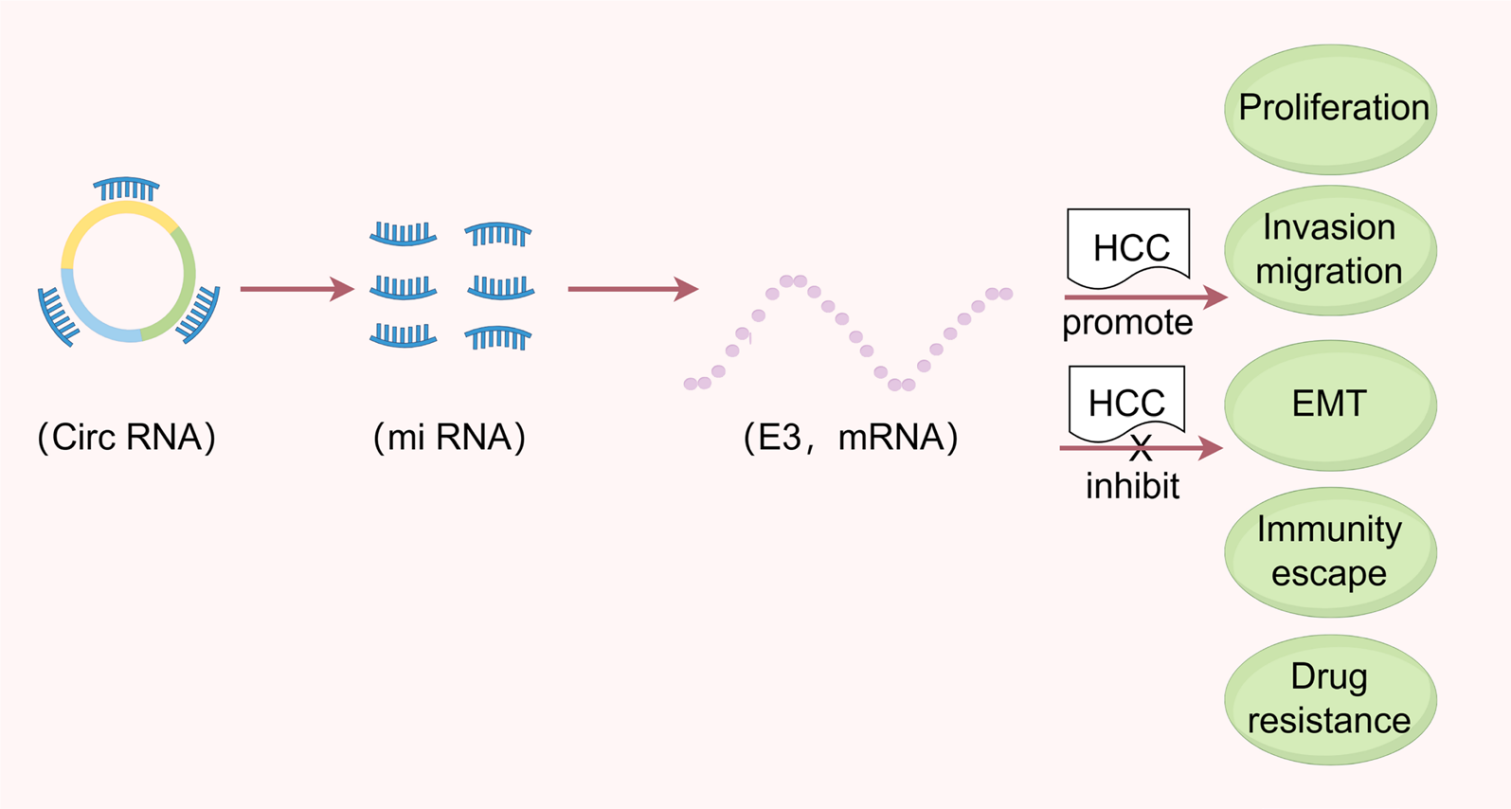

**Supplementary Information:**

The online version contains supplementary material available at 10.1186/s40001-024-01893-6.

## Introduction

Liver cancer remains a global health challenge, with an estimated incidence of > 1 million cases by 2025. HCC is the most common form of liver cancer and accounts for ~ 90% of cases [1, 2]. It is ranked third in global cancer mortality and represents more than 8% of all cancer-related fatalities [3]. The causes of HCC include long-term infections with the hepatitis B and hepatitis C viruses, metabolic liver diseases, and alcoholism, which contribute to somatic genetic variation and epigenetic modifications and ultimately result in hepatocarcinogenesis [4]. Despite advancements that have been made in treatment, those diagnosed with HCC, particularly in China, only have a 14.1% 5-year survival rate [5]. As a result, identifying subgroups of HCC with therapeutic relevance and new predictive biomarkers or signatures is imperative for improved risk classification and personalized treatment [6].

CircRNAs are non-coding RNAs that occur due to the back-splicing of pre-mRNA [7]. The essential functions circRNAs perform include acting as miRNA sponges and regulating protein translation [8, 9]. Our previous research showed circRNAs to be involved in several characteristics, including cell aggression and immune infiltration [10, 11]. It was discovered that Circ 0061984 (circPTTG1IP) functions as a competitive endogenous RNA (ceRNA) by binding to miR-16-5p, which increases the expression of the E3 ubiquitin ligase RNF125 [12]. Sun et al. discovered that circ-ADD3 inhibits HCC metastasis through the promotion of EZH2 degradation through CDK1-mediated ubiquitination [13]. One study found that circ 0026134 acts as a sponge for miR-127-5p to upregulate TRIM25 and IGF2BP3 in HCC, which promotes HCC development and metastasis [14]. However, the direct relationship between circRNA and E3 ligase in the ceRNA network remains unclear.

The ubiquitin–proteasome system (UPS) is a crucial mechanism in cells that helps maintain protein homeostasis through the regulation of the degradation of unwanted or damaged proteins [15]. Ubiquitin is a modified molecule of 76 amino acids extensively conserved across eukaryotic cells that covalently binds and labels target substrates by a cascade reaction that involves ubiquitin-activating enzyme (E1), ubiquitin-conjugating enzyme (E2), and ubiquitin-protein ligase (E3) [16]. The ubiquitin-protein ligases (E3) are essential enzymes in the UPS that are responsible for the regulation of several common HCC signaling pathways [17]. A growing amount of evidence has suggested that several structural or functional abnormalities in E3 ligases contribute to HCC development [17]. However, further research is required in order for the relationship between E3 ubiquitin ligase and HCC to be fully understood. Further investigation of the mechanisms of E3 ligase dysfunction in HCC could also offer new insights into the disease while also promoting the development of novel treatment approaches.

In this research, a nomogram and CRE3UL signature were developed as a means of enhancing the capacity to evaluate the prognosis and therapeutic responses of HCC patients. CRE3UL was created by combining the expression data and clinical information of HCC patients. Using gene set variation analysis (GSVA), the functions of gene sets associated with cancer hallmarks and their relationships to the prognoses of HCC patients were thoroughly investigated. A CRE3UL signature was created for evaluating the prognosis and treatment responsiveness of HCC patients. A nomogram for predicting the prognoses of HCC patients was then created using this signature and clinical data. Finally, experimental research was conducted to deepen the connection between E3 ubiquitin ligase signature and HCC.

## Materials and methods

### Tissue samples and pertinent clinical information

Twenty-four patients with primary HCC at Northern Jiangsu People’s Hospital’s Department of Hepatobiliary Pancreatic Surgery were identified and underwent surgical therapy. Twenty-four pairs of HCC and neighboring tissue samples were gathered from the patients in the hospital. This research was conducted in accordance with the guiding principles of the Declaration of Helsinki. Sample donors all signed informed consent forms.

### Data downloading and processing

The mRNA expression data and clinical information for three independent HCC cohorts were downloaded from The Cancer Genome Atlas (TCGA), the International Cancer Genomics Consortium (ICGC), and the Gene Expression Omnibus (GEO). The circRNA expression data were acquired from GSE97332 (371 HCC and neighboring tissues) and GSE94508 (50 HCC and neighboring tissues) in the GEO database. Data collection was performed in accordance with the usage guidelines of the TCGA, ICGC, and GEO databases. For data normalization, the values of the fragments per kilobase of transcript per million (FPKM) data (FPKM) in the three RNA-seq cohorts were transformed into transcripts per million kilobase (TPM) values using the R package “limma” [18]. Data that lacked complete clinical information or patients with an overall survival time of < 30 days were excluded due to other potential mortality causes [19].

### Identification of genes with differential expression

The R “limma” package was utilized to find differentially expressed circRNAs (DEcircRNAs). The “edgeR” package in R was utilized for evaluating differentially expressed mRNAs (DEmRNAs) and miRNAs (DEmiRNAs). The cut-off values of differentially expressed genes (DEGs) were |log2-fold change (FC)|> 1.5 and *p* < 0.05.

### Constructing the competing endogenous RNA network

The target miRNAs of DEcircRNAs were obtained by querying the Cancer-Specific CircRNA Database (CSCD), after which the overlapping target DEmiRNAs were obtained by intersecting with DEmiRNAs [20]. The miRDB and TargetScan databases were then used to anticipate the identified target mRNAs of the DEmiRNAs. Overlapping differentially expressed targeted E3 ubiquitin ligases were obtained by the intersection of targeted mRNAs, DEmRNAs, in addition to differentially expressed E3 ubiquitin ligases [21, 22]. Finally, Cytoscape was used to visualize the ceRNA network, which consisted of circRNA–miRNA–mRNA [23].

### Functional enrichment analysis

R package “clusterProfiler” and the KOBAS (KEGG Orthology-Based Annotation System) database were used to conduct Gene Ontology (GO) and Kyoto Encyclopedia of Genes and Genomes (KEGG) analysis for studying the biological mechanism of CRE3UL signature regulation [24].

### Consensus clustering

Unsupervised clustering was performed on all the HCC patients from TCGA using the “ConsensusClusterPlus” package for identifying distinct molecular patterns linked with diverse overall survival (OS) outcomes based on prognostic gene sets [25]. This research determined k values where the cumulative distribution function (CDF) plots stabilized at a maximum and the consensus matrix had diagonal blocks that were relatively prominent. The optimal clustering number was then established, which was validated using the proportion of the ambiguous clustering (PAC) method [26, 27].

### Analysis of immunological function and immunological infiltration

GSVA was undertaken as a means of exploring the Hallmark pathway of HCC patients in ubiquitin (UB) clusters A and B. The stromal score, immune score, estimate score, and immune cell infiltration in two clusters were all examined using R packages “ESTIMATE” and “CIBERSORT”. The relative levels of infiltration by 23 immune cells in HCC samples were determined using the Single-Sample Gene Set Enrichment Analysis (ssGSEA) method [28]. Finally, immune-checkpoint-related genes were analyzed to assess the immune treatment responses of UB cluster A and B HCC patients.

### Construction and verification of the CRE3UL signature

342 HCC patients in the TCGA cohort were randomly divided into the training set (*n =* 206) and testing set (*n =* 136) with a ratio of 6:4 [29]. Univariate Cox regression was used for screening significant univariate genes in the training cohort. In the training cohort, these relevant univariate genes were then subjected to LASSO Cox regression analysis to minimize the risk of overfitting between signatures [30, 31]. The five genes for building risk models were further selected using multivariate Cox regression, and their correlation coefficients were then computed. Internal validation was performed using the testing set and the complete TCGA cohort to validate the predictive efficacy of the model. The ICGC-LIRI cohort was used as external validation and time-dependent ROC analysis (t-ROC) was used to assess the sensitivity and specificity of the signature. The accuracy and discrimination of the prognostic risk model were compared with clinical characteristics. Finally, Principal Component Analysis (PCA) was used to further validate the capacity to group of the signature.

### Development and assessment of the nomogram

R package “rms” was used to generate a nomogram including clinical characteristics and a risk score. The calibration curves for years 1, 2, and 3 were also drawn to validate the accuracy of the nomogram. The survival for progression-free survival (PFS) in HCC patients of the Kaplan–Meier integrated nomogram was then analyzed.

### Assessing the tumor microenvironment (TME) and tumor mutation burden (TMB) correlation

Analyzing somatic mutations to evaluate the Tumor Mutational Burden scores. R package “maftools” was used to generate waterfall graphs for the high- and low-risk groups. GSVA was used to study KEGG pathways in the high- and low-risk patients. In addition, the ssGSEA method was used to determine the relative levels of infiltration by 28 immune cells in HCC samples.

### Immunotherapy drug sensitivity and effectiveness evaluation

R package “pRRophetic” was utilized for estimating chemotherapeutic sensitivity by measuring the 50% highest inhibitory concentration (IC50) of distinct groups of samples using ridge regression [32]. Immunotherapy response was subsequently predicted using the Tumor Immune Dysfunction, Exclusion, MSI Expr Sig, and TIDE Tool. Immune checkpoint-related genes were then used to assess the immune treatment responses of HRG and LRG HCC patients. Higher expression levels of genes associated with immunological checkpoints suggested the suitability of patients for immunotherapy. Immune Profile Score (IPS) analysis was then used as a means of evaluating the immunogenicity of both groups.

### Quantitative real-time PCR

Total RNA was isolated using the TRIzol reagent (Invitrogen Carlsbad, CA, USA) according to the manufacturer’s instructions from 24 matched HCC and paracancerous tissues. Reverse transcription of cDNA was subsequently performed using the PrimeScriptTM RT reagent Kit (Takara Bio Inc., Japan). The SYBR-Green PCR kit (Takara, Osaka, Japan) was used in a Rotor-Gene 3000 machine (Corbett Life Science, Sydney, Australia) for conducting real-time PCR. Finally, the 2^-ΔΔCt^ method was utilized to perform data analysis.

### Single-cell sequencing analysis

Tumor Immune Single Cell Hub 2 (TISCH2) is a single-cell RNA-seq database that enables interactive single-cell transcriptome visualization of the TME while also providing specific annotation of cell types [33]. The LIHC_GSE166635 dataset in TISCH2 is a liver cancer dataset that is generated using the 10 × Genomics platform. It contains single-cell RNA-sequencing data for 10 liver cancer samples and 6 normal liver tissue samples [34].

### Statistical analysis

Kaplan–Meier and log-rank tests were used for evaluating the OS rates of HCC patients. The R 4.1.2 and GraphPad Prism 8 were used for conducting statistical analyses and *P* < 0.05 indicated a statistically significant difference.

## Results

### Differentially expressed genes and the competing endogenous RNA network in HCC patients

**Fig. 1:**
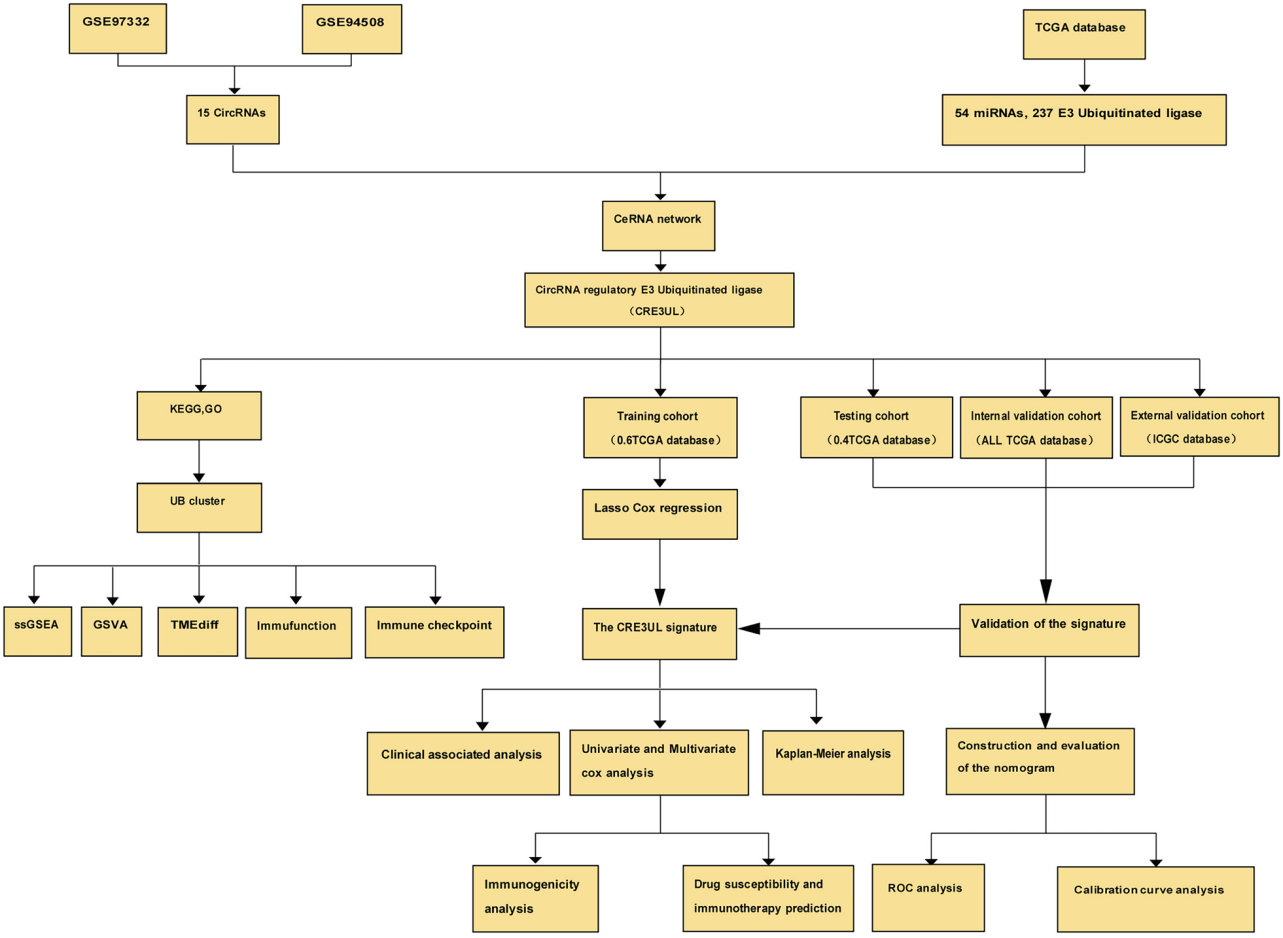
Flowchart of the analysis

**Fig. 2:**
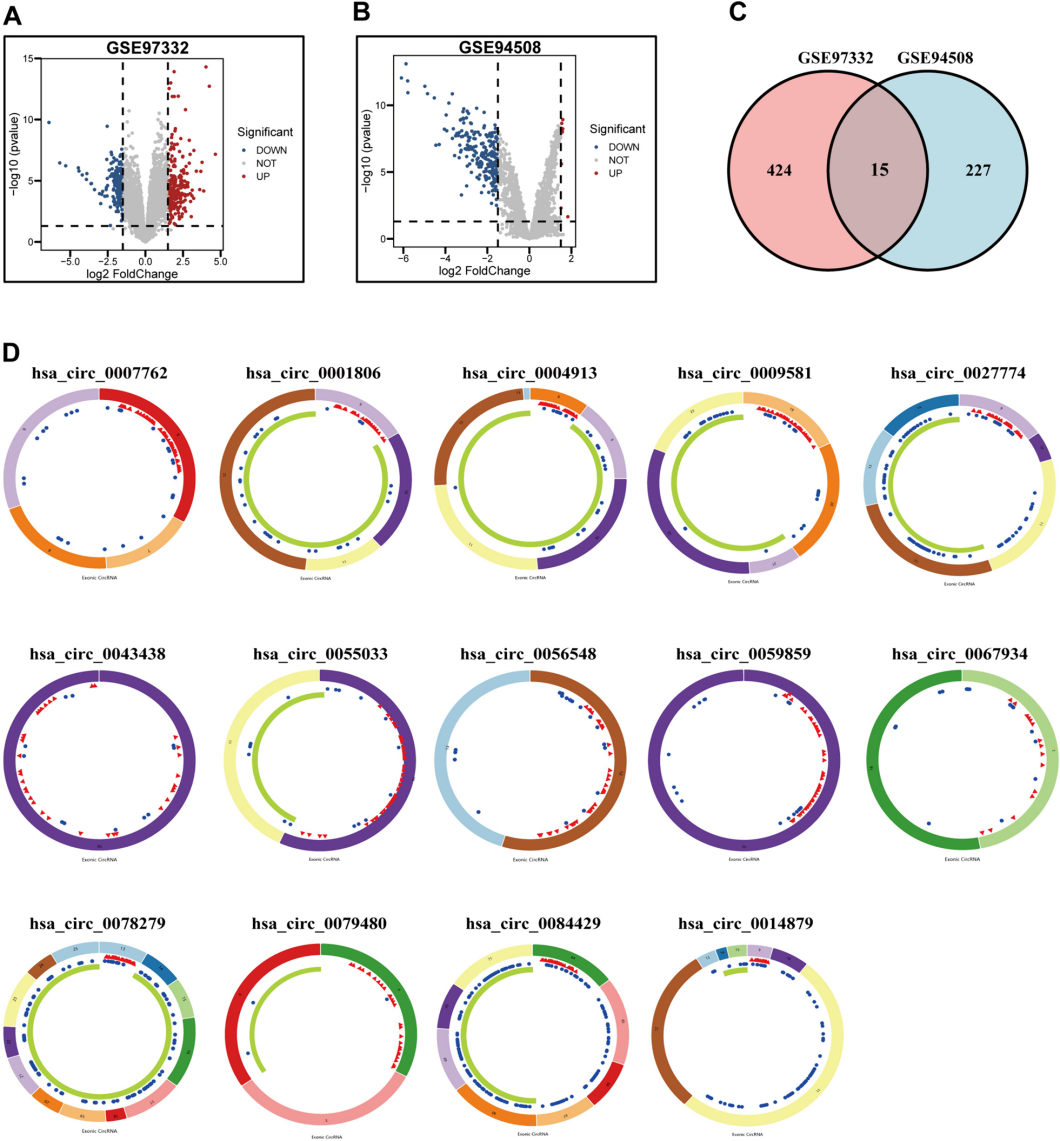
**A** Volcano plot of differentially expressed E3 ubiquitin ligase from GSE97332. **B** Volcano plot of differentially expressed E3 ubiquitin ligase from GSE94508. **C** Venn diagram to identify differentially expressed E3 ubiquitin ligase. **D** Interaction patterns of the 14 differentially expressed circRNAs in HCC. Red, blue, and green represent microRNA response elements, RNA-binding proteins, and open reading frames, respectively

**Fig. 3:**
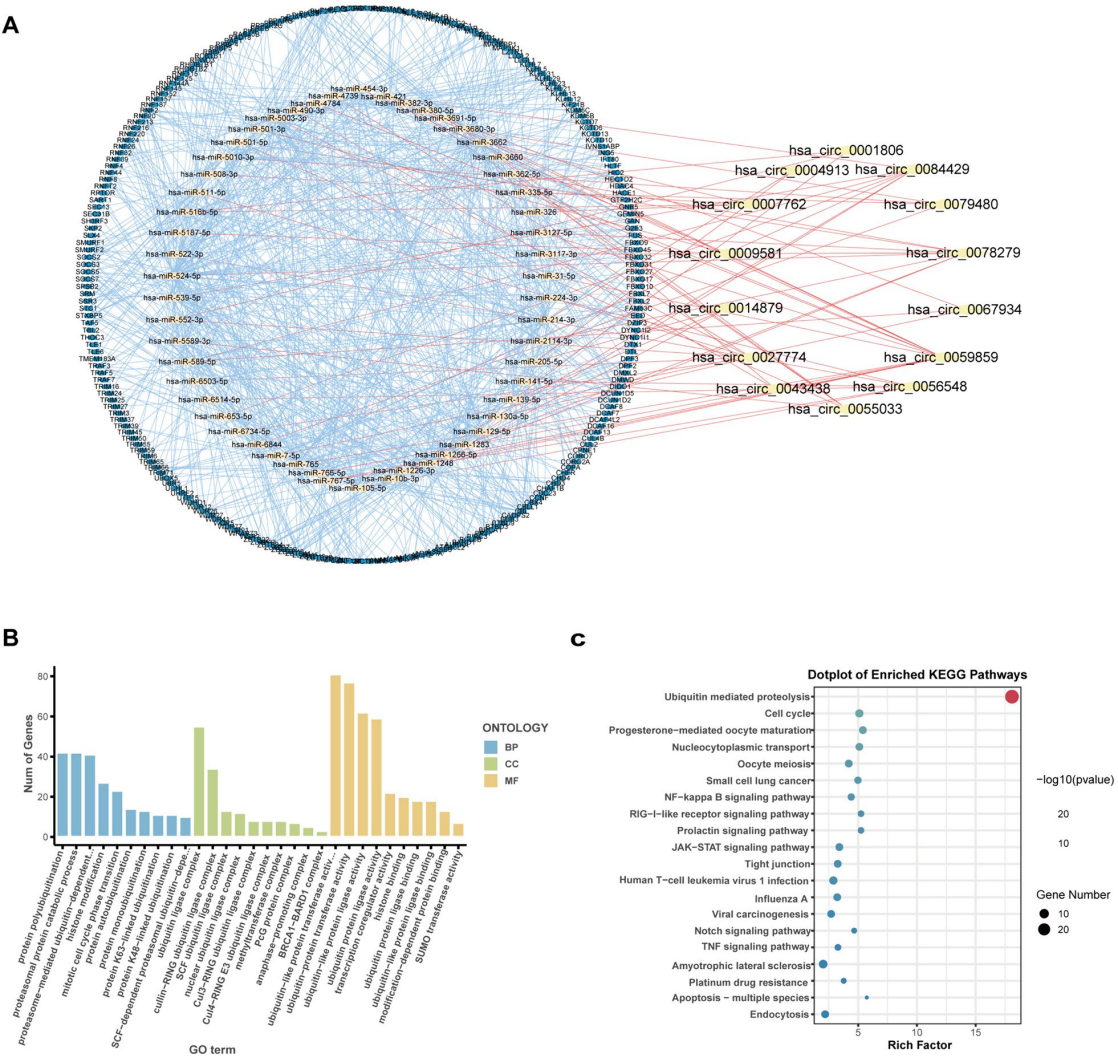
Functional enrichment analysis of CRE3UL. **A** circRNA–miRNA–mRNA (E3) ceRNA network in CRE3UL. Red, yellow, and blue nodes indicate 14 DEcircRNAs, 54 DEmiRNAs, and 237 DEmRNAs (E3), respectively. **B** GO enrichment analysis. **C** KEGG enrichment analysis. CRE3UL, circRNA-regulated E3 ubiquitin ligase; ceRNA, competing endogenous RNA; GO, Gene Ontology; KEGG, Kyoto Encyclopedia of Genes and Genomes

The workflow of this study is illustrated in Fig. 1. 15 DEcircRNAs in HCC tissues were obtained from the GSE97332 and GSE94508 datasets (|log2FC|> 1.5 and p < 0.05) (Fig. 2A–C). However, only 14 circRNAs were found in the database (Fig. 2D). 300 DEmiRNAs and 6,565 DEmRNAs in HCC tissues were obtained from the TCGA database. Next, we obtained 897 E3 ubiquitin ligases from the IUUCD (http://iuucd.biocuckoo.org/). However, only 833 E3 ubiquitin ligases were extracted from the TCGA and ICGC databases. The 6565 DEmRNAs in the TCGA database were then intersected with 833 E3 ubiquitin ligases to obtain an overlapping total of 344 differentially expressed E3 ubiquitin ligases. Furthermore, the CSCD was used for predicting the target miRNAs of 14 circRNAs and 676 target miRNAs were obtained. The 300 DEmiRNAs in the TCGA database were then intersected with 676 target miRNAs, which resulted in an overlapping of 54 target DEmiRNAs. The miRDB and TargetScan databases were used for predicting the targeted mRNAs of the 54 target DEmiRNAs, which yielded 776 targeted mRNAs. By intersecting the 776 targeted mRNAs with the 344 differentially expressed E3 ubiquitin ligases, an overlapping total of 237 differentially expressed targeted E3 ubiquitin ligases was obtained. Finally, the CRE3UL ceRNA network consisting of 14 DEcircRNAs, 54 DEmiRNAs, and 237 DEmRNAs (E3) was constructed (Fig. 3A).

### CircRNA-regulated E3 ubiquitin ligase biological function analysis

Functional enrichment analyses were performed to elucidate the biological roles of circRNA-regulated E3 ubiquitin ligases. The biological process (BP) included protein polyubiquitination and proteasomal protein catabolic process, the cellular component (CC) included the ubiquitin ligase complex, and the molecular function (MF) included ubiquitin-like protein transferase activity and ubiquitin-protein transferase activity (Fig. 3B). KEGG analysis showed the signature genes to be connected with the ubiquitin-mediated proteolysis signaling pathways (Fig. 3C).

### CRE3UL-guided identification of UB clusters with prognostic significance

**Fig. 4:**
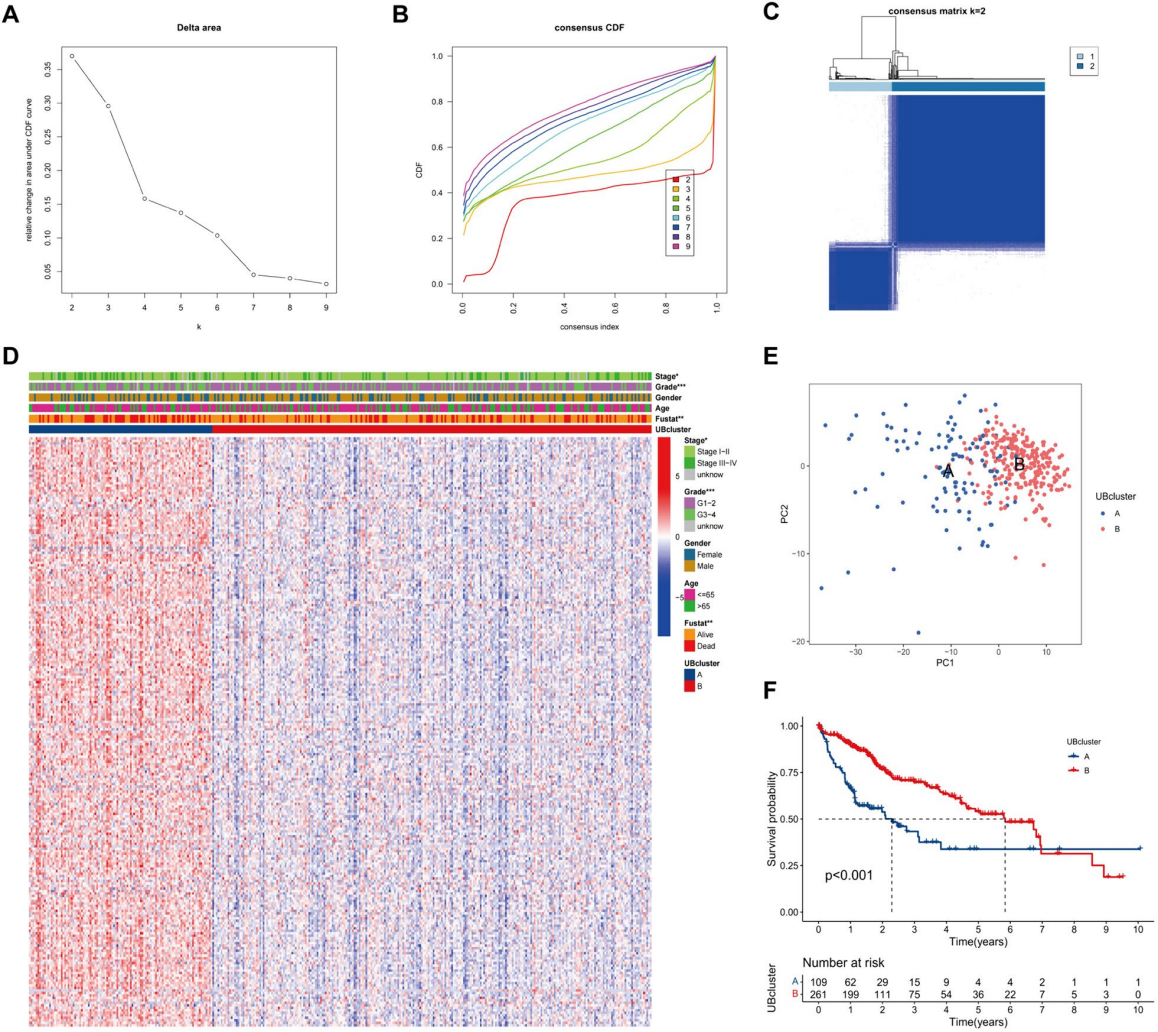
Identification of CRE3UL-based UB cluster. **A** The corresponding relative change in area under the cumulative distribution function (**C**, **D**, **F**) curves and the optimal number of clusters (k) was 2. **B** Consensus clustering CDF for *k* = 2 to 9. **C** Heatmap of sample clustering at consensus *k* = 2. **D** Heatmap showing the GSVA score of stage, grade, gender, age, and fustat in two UB clusters. **E** PCA plot visualizing the two UB clusters **F** Kaplan–Meier survival plots of cluster 1 and cluster 2 for OS. OS, overall survival. PCA, principal component analysis. GSVA, Gene set variation analysis

Unsupervised consensus clustering of CRE3UL was performed and it was classified into distinct molecular clusters. The findings showed the optimum number of clusters to be two, which resulted in the CDF plots stabilizing at a maximum and the consensus matrix having a relatively prominent diagonal block. The PAC algorithm further corroborated this (Fig. 4A–C). The CRE3UL was ultimately categorized into two separate clusters (Fig. 4D and E). Notably, Kaplan–Meier curve analysis showed a significantly better prognosis for UB cluster B than for UB cluster A (Fig. 4F).

### Relationship between CRE3UL-based UB clusters and tumor microenvironment, immunological infiltration, immune function, and immune checkpoint

ssGSEA was used in the whole TCGA cohort to delineate the infiltration of the immune cells to learn more about the potential clinical benefits of UB clusters. As can be seen in Supplementary Fig. 1A, UB cluster A was substantially connected with infiltrating immune cell types, including activated CD4 T cell, T follicular helper cell, and type 2 T helper cell. GSVA analysis showed that UB cluster A was substantially concentrated in processes linked to carcinogenic pathways, including the MYC, WNT, TGF, and PI3K signaling pathways, and UB cluster B was mainly found to be concentrated in processes linked to material metabolism, including the cholesterol homeostasis, fatty acid metabolism, and xenobiotic metabolism (Supplementary Fig. 1B). In addition, the violin plot showed UB cluster B to exhibit higher Stroma, Immune, and ESTIMATE Scores (*P* < 0.05, Supplementary Fig. 1C). Immune function was evaluated using the ssGSEA methodology and type II and type I interferon responses showed that individuals in UB Cluster B exhibited a higher level of cytolytic activity (Supplementary Fig. 1D). Due to the role of immune-checkpoint inhibitors (ICIs) in HCC therapy, UB clusters A and B were analyzed for immunological checkpoint gene expression. The majority of immune checkpoints, including CD-274, PDCD-1 (PD-1), and CTLA4, were found to be expressed at a greater level in UB cluster A, which demonstrated that patients in UB cluster A would be resistant to immunotherapy (Supplementary Fig. 1E).

### Development and validation of the CRE3UL signature

**Fig. 5:**
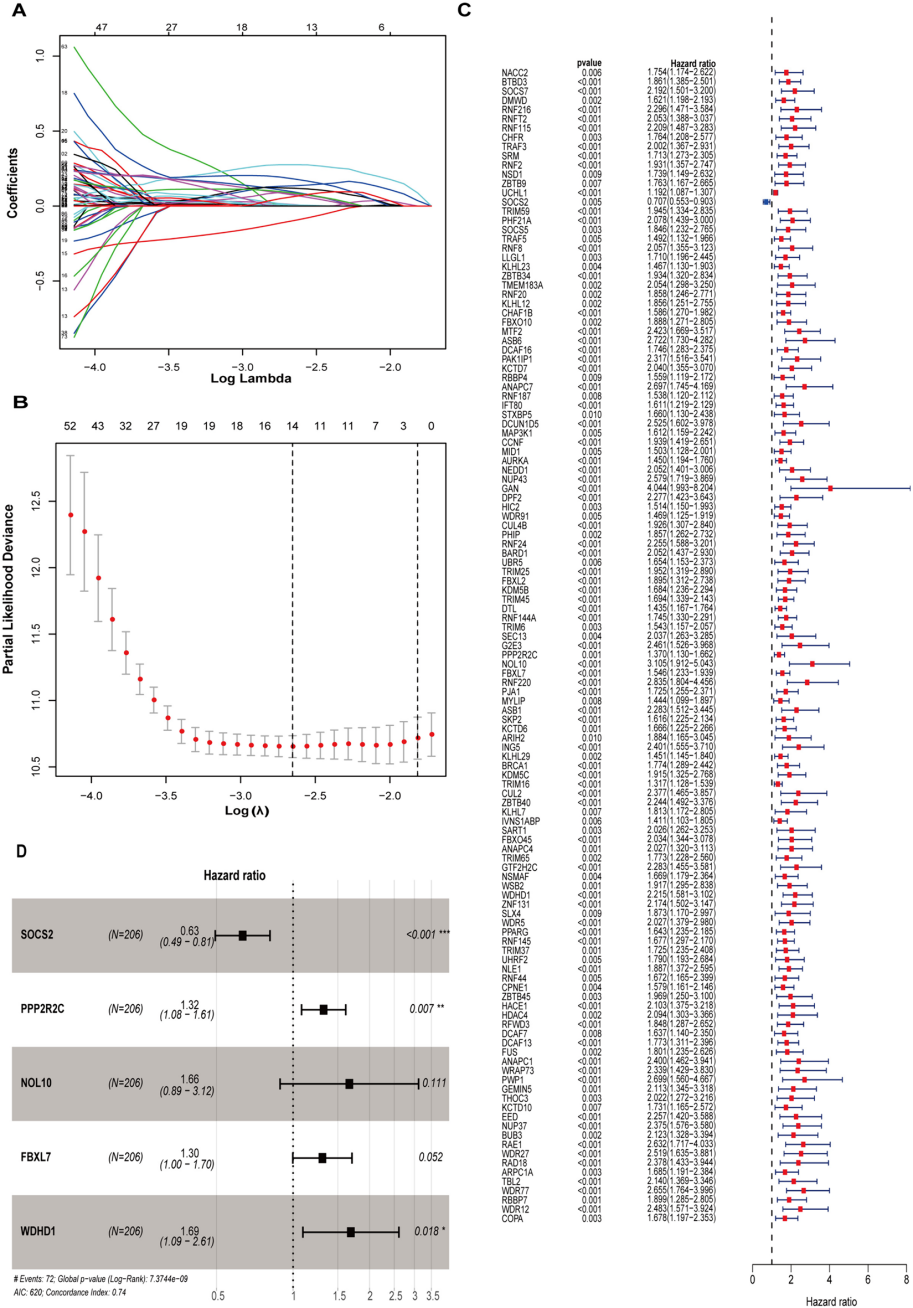
Establishment of the circRNA regulatory E3 ubiquitin ligase prognostic signature for HCC patients. **A**, **B** Cross-validation for tuning the parameter selection in the LASSO regression. **C** Univariate Cox analysis of the genes selected by circRNA. **D** The presentation of five E3 ubiquitin ligases in multivariate Cox regression analysis

Next, samples from 342 and 229 HCC patients were obtained from the TCGA and ICGC databases. The whole TCGA cohort was randomly split about 6:4 into a training dataset (*n =* 206) and a validation dataset (*n =* 136) (Supplementary Table 1). Additionally, the whole TCGA and ICGC cohort further served as internal and external validations. LASSO regression and Cox proportional hazard model analysis were used for identifying the most appropriate model in the training dataset (Fig. 5A and B). Following a univariate Cox analysis, 102 differentially expressed E3 ubiquitin ligases related to overall survival (OS) were chosen (Fig. 5C). In addition, multivariate analysis identified the model formed by five E3 ubiquitin ligase risk scores, which were calculated through the use of the following formula: Riskscore = − 0.45 * EXP SOCS2 + 0.27 * EXP PPP2R2C + 0.50 * EXP NOL10 + 0.26 * EXP FBXL7 + 0.52 * EXP WDHD1) (Fig. 5D). HCC patients from different cohorts were separated into the HRG and LRG using the median risk score (Supplementary Fig. 2A and B). In various datasets, Kaplan–Meier analysis showed high-risk patients to have considerably lower OS rates than low-risk patients (Supplementary Fig. 2C). In addition, the t-ROC plot was used to assess the accuracy of the model. The area under the ROC curve (AUC) of each dataset in the 1-year, 2-year, and 3-year survival can be seen in Supplementary Fig. 2D.

### Subgroup analysis and Cox analyses of the CRE3UL signature

**Fig. 6:**
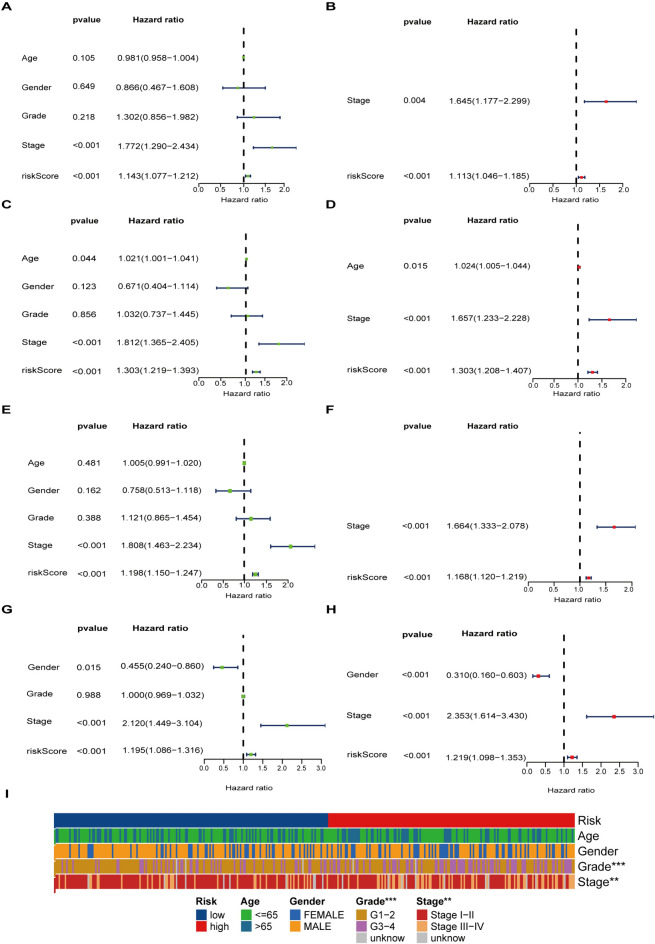
Univariate and multivariate Cox analyses of HCC. Univariate Cox regression results in the **A** TCGA training set, **C** TCGA testing set, **E** whole TCGA cohort, and **G** ICGC cohort. Multivariate Cox regression results in the **B** TCGA training set, **D** TCGA testing set, **F** whole TCGA cohort, and **H** ICGC cohort. **I** Relationship between the CRE3UL signature and clinical characteristics (***p < 0.001, **p < 0.01, *p < 0.05)

The CRE3UL signature was used for predicting the prognostic indicator for subgroups of TCGA database patients with different clinical features which can be seen in Supplementary Fig. 3. Regarding age, gender, grade, and clinical stage, the HRG exhibited lower OS rates than the LRG (*P* < 0.001). Univariate and multivariate Cox analyses found the CRE3UL signature to be an independent prognostic risk factor in the merged dataset. In the TCGA training set, univariate Cox regression analysis found the hazard ratio (HR) of the model to be 1.143 and a 95% CI of 1.077–1.212 (*P* < 0.001) (Fig. 6A). Multivariate Cox regression analysis then found the hazard ratio (HR) of the model to be 1.113 and a 95% CI of 1.046–1.185 (*P* < 0.001) (Fig. 6B). In TCGA testing, whole TCGA, and ICGC cohorts, univariate Cox regression analysis yielded respective HR values of 1.303 (95% CI 1.219–1.393, *P* < 0.001), 1.198 (95% CI 1.150–1.247, *P* < 0.001), and 1.195 (95% CI 1.086–1.316, *P* < 0.001) (Fig. 6C, E, and G). Multivariate Cox regression analysis then yielded respective HR values of 1.303 (95% CI 1.208–1.407, *P* < 0.001), 1.168 (95% CI 1.120–1.219, *P* < 0.001), and 1.219 (95% CI 1.098–1.353, *P* < 0.001) (Fig. 6D, F, and H). In addition, the relationship between CRE3UL and clinicopathologic features was investigated, and it was found that tumor, grade, and stage were significantly associated with CRE3UL **(**Fig. 6I**)**.

### Comparing the accuracy and discrimination of the prognostic risk model with clinical characteristics

To evaluate the credibility of the model, candidate predictive factors, including tumor stage, age, gender, and pathological grade, were used to assess the ability of the risk score model to see whether patient survival could be accurately predicted. In addition, the 1-year prognosis AUC curve and the C-index were assessed. The risk score had the highest AUC values compared to other factors (Supplementary Fig. 4A). Similarly, the C-index of the risk score was consistently higher than other clinical indicators over time (Supplementary Fig. 4B), which suggests a more accurate forecasting of the model when evaluating HCC prognosis. Moreover, the risk scores were significantly higher in tumor grades 3–4 (*P* < 0.001) or tumor stages III–IV (*P* < 0.001) when the relationship between risk scores and clinical features of HCC patients was monitored (Supplementary Fig. 4C and D). However, no notable relationship was observed between risk score and gender or age. (Supplementary Fig. 4E and F).

### Principal component analysis (PCA) further confirms the grouping capability of the signature

PCA was performed with TCGA and ICGC genome expression profiles of five E3 ubiquitin ligases to discover the differences between the HRG and LRG. The relative distribution of five E3 ubiquitin ligases in the HRG and LRG based on TCGA and ICGC genome expression profiles can be seen in Supplementary Fig. 5A and, B. Next, t-distributed stochastic neighbor embedding (t-SNE2) was used to determine the distinctions between the HRG and LRG. The model showed distinct distributions for LRG and HRG (Supplementary Fig. 5C and D). The above findings show that the prognostic model was able to differentiate distinctly between the two groups.

### Development and assessment of the nomogram

**Fig. 7:**
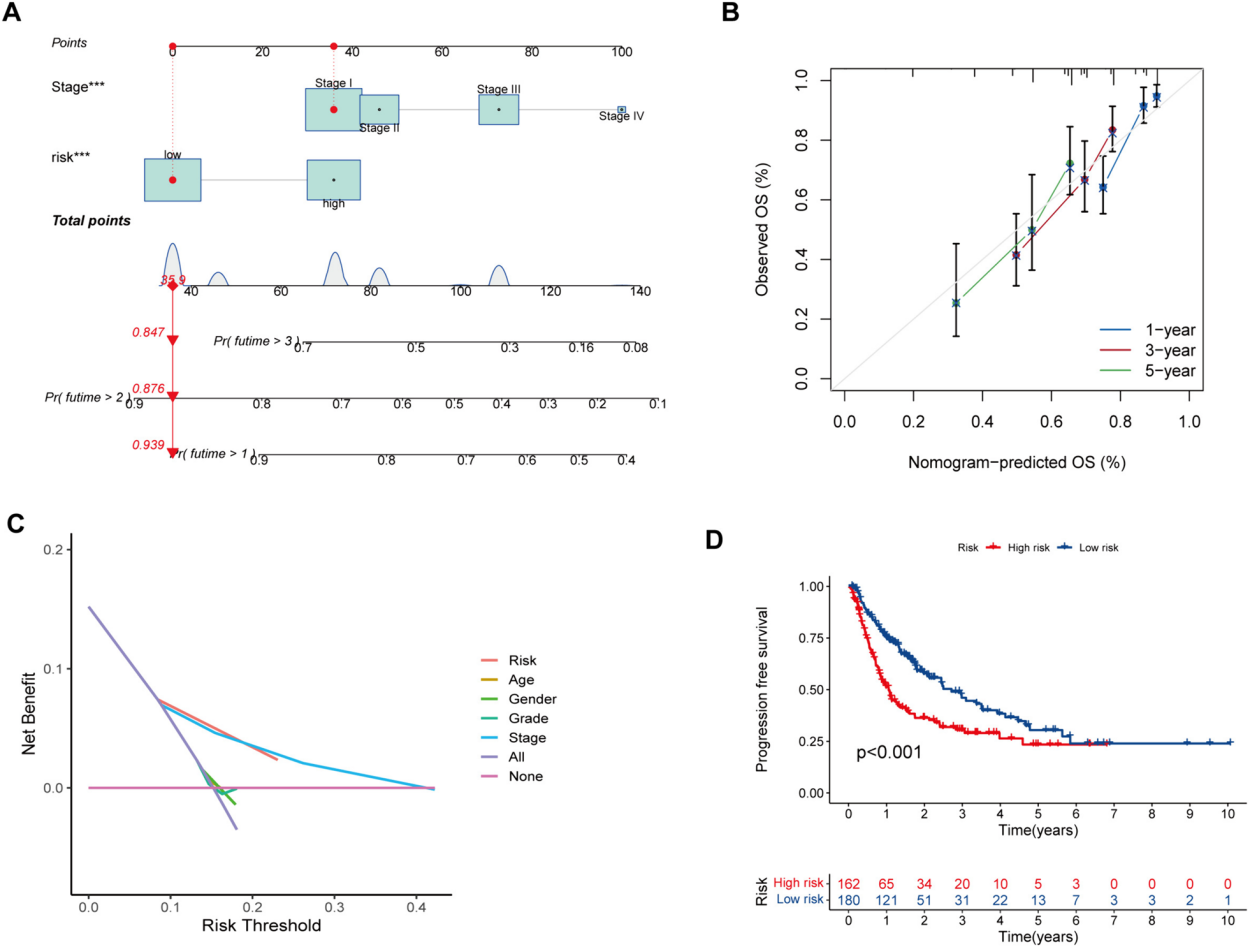
Establishment and assessment of a nomogram in the entire set. **A** The nomogram predicts the probability of the 1, 2, and 3 years of OS. **B** The calibration plot prediction via nomogram of the OS at 1, 2, and 3 years. **C** Decision curve analysis for the nomogram, age, gender, grade, stage, and risk score. **D** Kaplan–Meier survival analysis of the integrated nomogram for PFS in HCC patients

The nomogram predicts 1, 2, and 3-year OS by combining the risk model and tumor stage information from the whole dataset (Fig. 7A). The calibration plots for 1-, 2-, and 3-year OS show that the predictions and actual observations of the nomogram have a good consistency (Fig. 7B). The generated nomogram outperforms a risk model alone in terms of prediction accuracy. In addition, decision curve analysis (DCA) was conducted to evaluate the clinical utility of the nomogram. The nomogram was found to outperform both the risk model and clinical features regarding net benefit (Fig. 7C). Finally, the Kaplan–Meier analysis for PFS demonstrated a significantly better prognosis for low-risk patients than high-risk patients (Fig. 7D). According to these results, the nomogram can more accurately predict the prognosis of HCC patients than a single clinical feature or our signature, which may facilitate easier clinical management and provide tailored treatment options.

### Relationship between the CRE3UL signature and somatic mutations

**Fig. 8:**
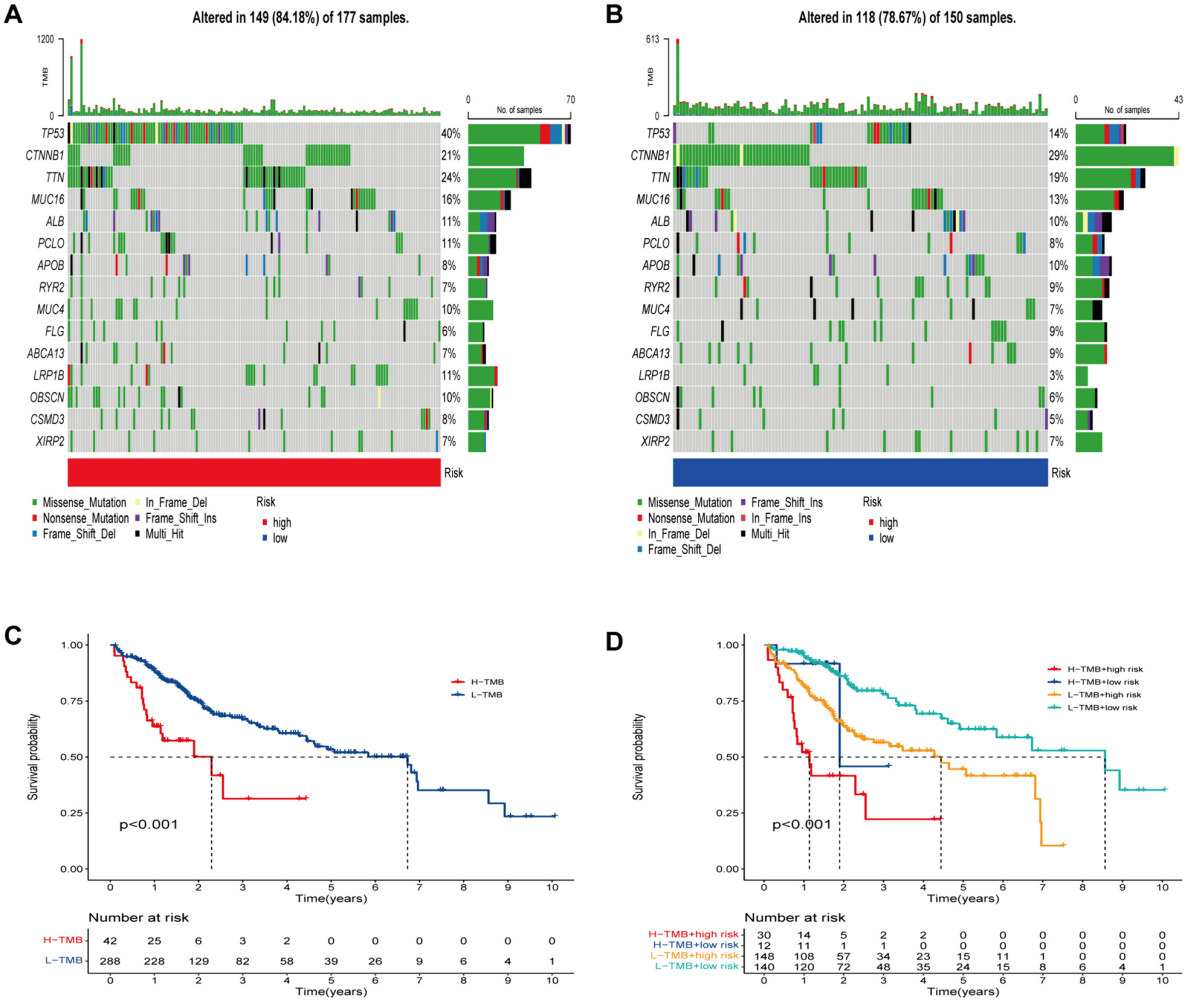
Relationship between the E3 ubiquitin ligase signature and somatic mutation. Waterfall plots of 15 genes with the highest mutation rates in the high-risk group (**A**) and the low-risk group (**B**). **C** Kaplan–Meier analysis of TMB in HCC patients. **D** Kaplan–Meier analysis of the correlation between risk score and TMB

Mutation accumulation typically drives tumor development and remodeling of the tumor immune microenvironment (TIME). Therefore, the difference between HRG and LRG regarding somatic mutations was investigated. In the HRG, TP53 (40%), TTN (24%), CTNNB1 (21%), and MUC16 (16%) were found to have the most mutation frequencies (Fig. 8A). In the LRG, CTNNB1 (29%), TTN (19%), TP53 (14%), and MUC16 (13%) were found to have the most mutation frequencies (Fig. 8B). The findings showed the HRG to have a greater number of immune-related mutations. The optimal TMB cutoff was then used to separate patients into low and high TMB groups. The findings showed that a higher TMB value was connected with a shorter OS (Fig. 8C, p < 0.001). Risk score and best TMB cut-off values were then used to separate TCGA patients into four groups. Patients with low TMB and risk levels were found to have a higher survival rate than those with high TMB and risk levels, as can be seen in the Kaplan–Meier analysis (Fig. 8D, p < 0.001).

### CRE3UL signature was associated with the immunological state in the HCC tumor microenvironment

**Fig. 9:**
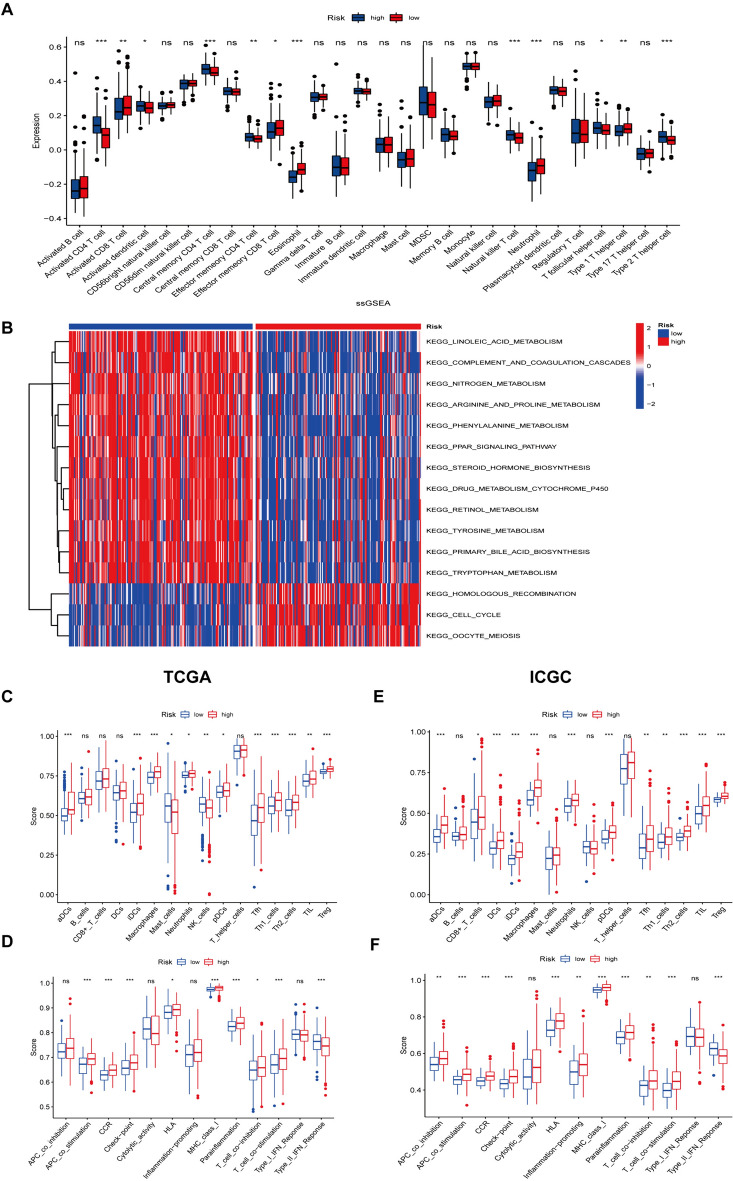
Overview of CRE3UL signature-related immune infiltration. **A** The box plot presents the relative composition of multiple cell types in the high-risk and low-risk groups of patients with the CRE3UL signature. **B** GSVA analysis of biological pathways between the two distinct risk groups. **C** Comparison of immune cell infiltration in the high- and low-risk groups in the TCGA-LIHC cohort. **D** Comparison of immune function in the high- and low-risk groups in the TCGA-LIHC cohort. **E** Comparison of immune cell infiltration in the high- and low-risk groups in the ICGC-JIHC cohort. **F** Comparison of immune function in the high- and low-risk groups in the ICGC-JIHC cohort. Statistical significance was denoted with *p < 0.05, **p < 0.01, and ***p < 0.001

The immunological microenvironment of patients from HRG and LRG was then explored. ssGSEA was used in the whole TCGA cohort to delineate the infiltration of the immune cells. As can be seen in Fig. 9A, the HRG was substantially linked with infiltrating immune cells, including Activated dendritic cell, Activated CD4 T cell, Central memory CD4 T cell, Effector memory CD8 T cell, Natural Killer T cell, T follicular helper cell, and Type 2T helper cell. GSVA analysis showed HRG to be substantially linked with homologous recombination, cell cycle, and oocyte meiosis. However, the LRG were predominantly linked with metabolism-related processes, including primary bile acid biosynthesis, phenylalanine, tyrosine metabolism, and linoleic acid metabolism (Fig. 9B). As previously stated, the findings demonstrate an apparent disparity in biological function between the HRG and LRG. Next, ssGSEA was used in the RNA-seq data of the TCGA-LIHC cohort to assess immune cell infiltration and associated function. It was found that the populations of immune cells that promote tumor-killing actions, such as NK cell and Mast cell, were linked with LRG patients, while immune cell populations that have an inhibitory influence on the tumor-killing action, such as regulatory T cells (Tregs), were linked with HRG patients (Fig. 9C). Furthermore, type II interferon responses showed that individuals in low-risk groups exhibited a higher level of cytolytic activity (Fig. 9D). At the same time, the result of the immunological microenvironment in the ICGC cohort was similar to that of the TCGA cohort (Fig. 9E and F). Generally, the findings revealed there to be a correlation between low-risk patient groups and an immunological milieu that promotes tumor killing.

### Drug susceptibility and immunotherapy prediction

How well the CRE3UL signature predicted the outcome of therapy for HCC was then examined. The pRRophetic algorithm was used for calculating the IC50 values of four anticancer (gefitinib, nilotinib, gemcitabine, and sorafenib) medications. The findings showed gefitinib and nilotinib therapy to be beneficial for HRG patients, while gemcitabine therapy was beneficial for LRG patients. The IC50 value of sorafenib was not statistically different between HRG and LRG (Supplementary Fig. 6A–D). In addition, the investigation indicated that HRG patients had lower dysfunction scores (distribution of dysfunction in TCGA-HCC, Supplementary Fig. 6E, *p* < 0.001). Exclusion and MSI Expr Sig scores were greater in HRG than LRG patients (Supplementary Fig. 6F and H), while the TIDE scores were the inverse result (Supplementary Fig. 6G). Regarding immunotherapy, expressions of PDCD1 (PD1), CTLA-4, and CD276 were higher in HRG, which indicates that these patients would be resistant to immunotherapy (Supplementary Fig. 6I). Finally, IPS analysis was used for analyzing the immunogenicity of the two groups. According to the findings, ips ctla4 neg pd1 neg and ips ctla4 pos pd1 neg scores were greater in the group with low expression. (Supplementary Fig. 6J–M). The above findings provide additional reference values for the development of individualized treatment strategies for HCC patients.

### Functional enrichment analyses of five E3 ubiquitin ligases and qRT-PCR verification of their expression in HCC

**Fig. 10:**
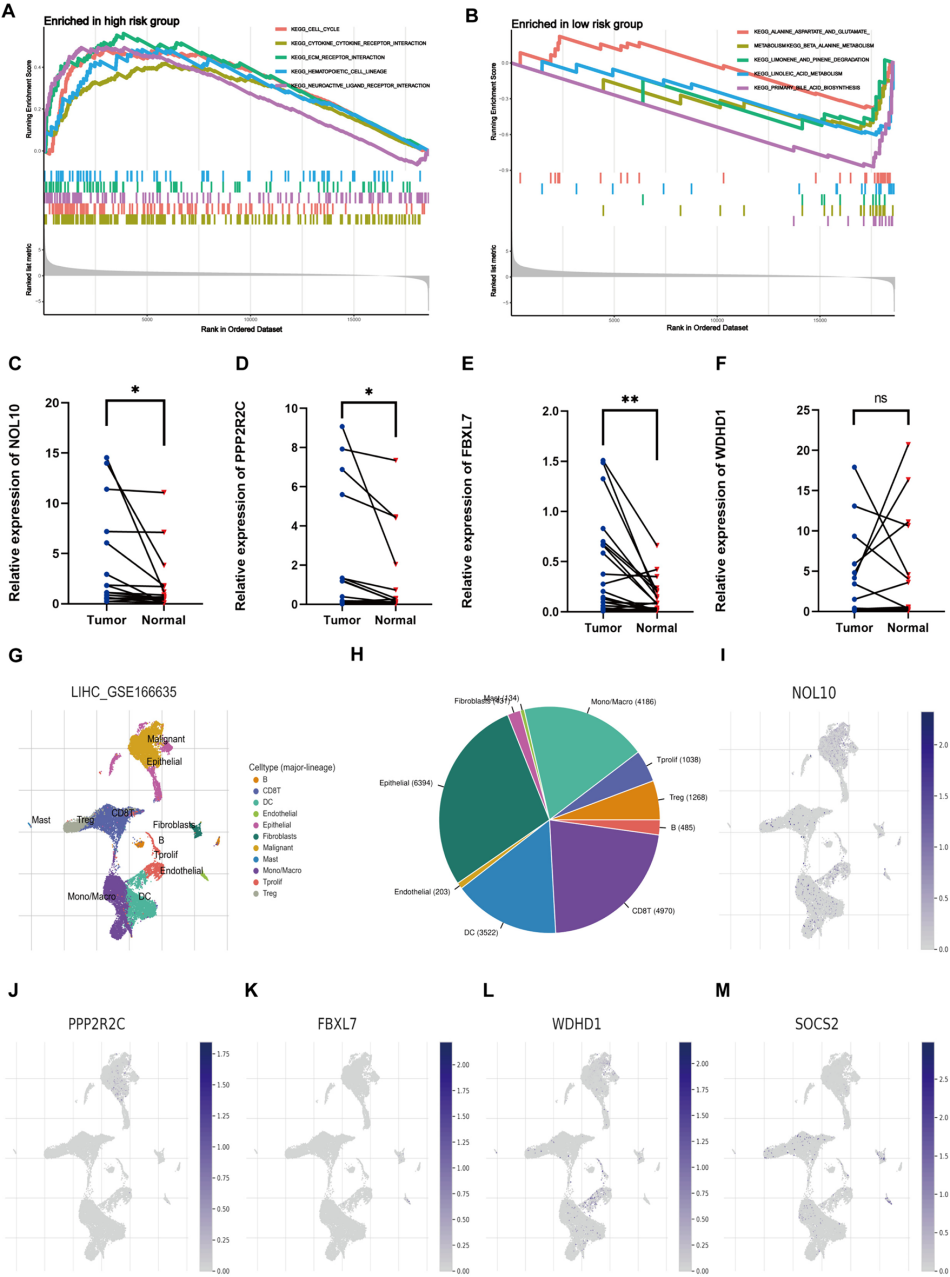
KEGG enrichment analysis for the high-risk (**A**) and low-risk groups (**B**) of CRE3UL. Validation of mRNA expression by real-time PCR and mRNA expression of five genes associated with E3 ubiquitin ligase in 24 HCC tissues and paracancerous tissues. ns, not statistically significant; *p < 0.05; **p < 0.01; ***p < 0.001 (**C–F**). **G**, **H** The cell types and their distribution in the GSE166635 dataset. **I–M** The distribution of five genes associated with E3 ubiquitin ligase in different cell types was analyzed using single-cell resolution in the GSE166635 dataset

Gene Set Enrichment Analysis (GSEA) was used for investigating the KEGG pathway enrichment in two groups. Cell cycle, Cytokine receptor interaction, and ECM receptor interaction pathways were substantially found to be linked with HRG patients (Fig. 10A). Similarly, Alanine aspartate and glutamate metabolism, Beta-alanine metabolism, Limonene, and pinene degradation pathways were found to be substantially enriched in LRG patients (Fig. 10B). qRT-PCR was then employed to assess the CRE3UL (NOL10, PPP2R2C, FBXL7, WDHD1, and SOCS2) expression in HCC. The mRNA expression levels in 24 HCC tissues and paracancerous tissues were evaluated. The findings revealed NOL10, PPP2R2C, and FBXL7 to be expressed more in HCC tissues than in paracancerous tissues, while SOCS2 showed the inverse results. However, the WDHD1 gene exhibited no great differences (Fig. 10C–F). The expression of SOCS2 in HCC tissues has been reported in existing literature studies [35]. Finally, TISCH2 was utilized for identifying the expression distribution of five E3 ubiquitin ligases at the single-cell level to investigate the TME heterogeneity. The GSE166635 dataset was divided into 11 types of cells (Fig. 10G). Figure 10H shows the number of cells in each cell type, with the distribution and number of various TME-related cells presented. Single-cell analysis showed that five E3 ubiquitin ligases expression was distributed in most immune cell types, which suggests that five E3 ubiquitin ligases are closely related to the TME in HCC (Fig. 10I–M).

### Discussion

HCC is susceptible to metastasis and has a poor prognosis [36]. Therefore, finding predictive prognostic biomarkers for HCC to improve the treatment results of HCC patients is essential. Increasing evidence indicates that E3 ligases play a crucial role in HCC by regulating the degradation of tumor promoters and repressors [17]. CircRNA-based signatures accurately predict the prognoses of cancer patients. However, it is uncertain if the CRE3UL signature can predict HCC prognosis.

This study focused on the CRE3UL signature. First, ubiquitin ligase-associated competitive endogenous RNA was constructed. CRE3ULs were established based on E3 ubiquitin ligase in the competitive endogenous RNA network. GO and KEGG analysis was then conducted on circRNA-regulated E3 ubiquitin ligases to elucidate their biological roles. Using GSVA, the functions of gene sets associated with cancer hallmarks and their relationships to the prognosis of HCC patients were thoroughly investigated. Two subgroups with differing prognostic outcomes were discovered. Next, two clusters were compared to the tumor microenvironment, immunological infiltration, immune function, and checkpoint. LASSO regression and Cox proportional hazard model analysis were used to identify the most appropriate model in the TCGA training dataset. Following univariate Cox analysis, 102 differentially expressed E3 ubiquitin ligases related to overall survival (*p* < 0.05) were chosen. Multivariate analysis also identified the model formed by five E3 ubiquitin ligase risk scores. According to ROC analysis and C-index, the model exceeded conventional clinical features in the prediction of HCC survival. A nomogram was used to evaluate the 1-, 2-, and 3-year overall survival rates of HCC patients, and the predicted and observed values were found to be consistent. As indicated by the C-index and DCA analyses, the created nomogram predicted the prognosis of HCC patients more accurately and reliably. The findings show that the model outperforms TNM staging. It has been proven that TMB is related to immunotherapy efficacy and prognosis for several different cancers. However, the precise mechanism of action in HCC was not identified [37]. Therefore, the somatic mutation differences between the two groups were compared. TP53 showed the greatest mutation rate (40%) in the HRG, while CTNNB1 exhibited the highest mutation rate (29%) in the LRG. According to previous results, the most commonly observed mutations in HCC are the TP53 and CTNNB1 genes [38, 39]. Exome sequencing studies surprisingly revealed CTNNB1 mutation to be substantially connected with alcohol-related HCC, while TP53 mutation was found to be prevalent in HBV-related HCC [40]. Theoretically, drinkers may be referred to the low-risk category, while more HBV-infected individuals may be referred to the high-risk category. This is consistent with the research findings. The immunological microenvironment of patients from HRG and LRG was subsequently analyzed. The populations of immune cells that promote tumor-killing actions, such as NK cell and Mast cell, were found to be linked with LRG patients, while immune cell populations that have an inhibitory influence on the tumor-killing action, such as regulatory T cells (Tregs), were found to be linked with HRG patients. Recent investigations have found HCC development and progression to be linked to a distinctive immune response profile of the liver microenvironment where CD4 + CD25 + Foxp3 regulatory T cells (Tregs) play a crucial role with their immunosuppressive function. Tregs are characterized by the expression of the transcription factor Foxp3 and various mechanisms ranging from cell-to-cell contact to secretion of inhibitory molecules have been implicated in their function. It is notable that Tregs amply express checkpoint molecules such as cytotoxic T lymphocyte-associated antigen 4 (CTLA4) and programmed cell-death 1 (PD1) receptor and represent a direct target of immune-checkpoint inhibitor (ICI) immunotherapy [41]. Regarding drug sensitivity prediction, the IC50 values of four anticancer (gefitinib, nilotinib, gemcitabine, and sorafenib) medications were calculated to predict how HRG and LRG patients would respond to anticancer drug therapy. Gefitinib and nilotinib treatment may be advantageous for HRG patients, but LRG patients may benefit from gemcitabine therapy, which demonstrates that the CRE3UL signature can be utilized to personalize the treatment of HCC patients. Immunotherapy response is generally predicted by immune-checkpoint molecule and IPS expression [42]. In this study, the high-risk patients had greater CTLA4, PD1, and PD-L1 expression levels and higher IPS values. Regarding immunotherapy, HRG patients were found to be better immunotherapy candidates than LRG patients. The aforementioned findings have revealed risk score to be a crucial indicator for the evaluation of immunological state. Recent studies have discussed the fundamental principles, effectiveness, and safety of Tyrosine Kinase Inhibitor (TKI) combined with Immune Checkpoint Inhibitor (ICI) treatment for hepatocellular carcinoma. These studies have also discussed the available results from other clinical trials that have used similar combinatorial therapeutic approaches [43]. The validation of the findings of this study can be enhanced by increasing the dataset through the collection of a larger sample size of hepatocellular carcinoma patients who are undergoing combined TKI + ICI therapy. The aim of this approach is to bolster the robustness of the results. Finally, qRT-PCR was employed for assessing the CRE3UL (NOL10, PPP2R2C, FBXL7, WDHD1, and SOCS2) expression levels in 24 HCC and paracancerous tissues. The findings revealed that NOL10, PPP2R2C, and FBXL7 were expressed more in HCC than paracancerous tissues, while SOCS2 showed the inverse result. However, the WDHD1 gene exhibited no great differences. This lack of significance could be attributed to the limited sample size and non-parallel protein and mRNA expressions.

Circ 0004913, which is a component of the developed E3 ubiquitin ligase-related ceRNA network for HCC, was downregulated in HCC tissues. High expression of circ 0004913 was found to be linked with a longer OS in all HCC patients [44]. One study has found that circ 0004913 sponges miR-1290 and regulates FOXC1 to inhibit the proliferation of HCC [45]. Zhou et al. found that circ 0001806 promotes HCC development through the miR-193a-5p/MMP16 axis [46]. Another study found the overexpression of circ 0067934 to be connected with a worse outcome in HCC patients [47]. However, there have been few investigations on other circRNAs in HCC and further research in this area is necessary. The risk prognostic model was subsequently established using LASSO and Cox regression analysis, based on five E3 ubiquitin ligases: SOCS2, NOL10, PPP2R2C, FBXL7, and WDHD1. SOCS2 (Suppressor of Cytokine Signaling 2) is a protein that is a member of the family of cellular regulators that is involved in negative feedback in cell signaling. The basic structure of SOCS2 includes an N-terminal domain, a central SOCS box, and a C-terminal tail, which enables its interaction with other signaling molecules for signal modulation. SOCS2 is one of the most critical circRNA-regulated E3 ubiquitin ligases in the network, and it has been found to be downregulated in HCC tissues and cells. Ren et al. discovered SOCS2 negatively regulated the activation of the JAK/STAT pathway in HCC cells, thereby inhibiting HCC progress [48]. Available research has shown SOCS2 overexpression to decrease HCC invasion and migration, while SOCS2 knockdown increases HCC development, which suggests that SOCS2 may limit the formation of HCC [49]. Furthermore, numerous investigations have suggested that SOCS2 also exerts significant effects in the central nervous system, metabolic regulation, immune response, mammary gland development, and other cytokine-dependent signaling pathways [50]. Another E3 ubiquitin ligase, NOL10 is a nucleolar protein, and its basic structure is likely to include domains that are associated with nucleolar functions. These domains may be involved in processes including ribosome biogenesis and RNA processing. Hu et al. discovered NOL10 to promote HCC migration and invasion. A higher expression of NOL10 has robust connections with poorer overall survival, relapse-free survival, post-progression survival, and disease-specific survival. ROC curves analysis has shown NOL10 to be a potential biomarker for HCC diagnosis with great sensitivity and specificity [51]. PPP2R2C, which is also known as Protein Phosphatase 2 Regulatory Subunit B, Gamma, is a subunit of protein phosphatase 2A (PP2A) that plays an important role in the regulation of cellular protein phosphorylation. Fan et al. discovered that PPP2R2C overexpression reduces the development of human glioma cells by inhibiting the mTOR pathway [52]. One study has revealed that MiR-572 promotes human ovarian cancer cell growth by inhibiting PPP2R2C expression [53]. Another study discovered that miR-1301 increases prostate cancer growth by directly targeting PPP2R2C [54]. In addition, F-box and leucine-rich repeat protein 7 (FBXL7), an F-box protein that is accountable for substrate recognition in the SKP1-Cullin-1-F-box (SCF) ubiquitin ligases, assumes a growing function in the control of tumorigenesis and tumor progression. FBXL7 induces polyubiquitylation and breakdown of various substrates and is involved in numerous biological processes, including apoptosis, cell proliferation, cell migration and invasion, tumor metastasis, DNA damage, glucose metabolism, planar cell polarity, and drug resistance [55]. FBXL7 exhibits aberrant expression in a variety of cancers. Patients who exhibited elevated levels of FBXL7 were found to demonstrate poorer OS across multiple cancer types, including colon adenocarcinoma, rectum adenocarcinoma, stomach cancer, liver cancer, thyroid cancer, lung cancer, and urothelial cancer. However, better survival outcomes were observed in kidney renal clear cell carcinoma, which suggests the oncogenic nature of FBXL7 [55]. WD repeat and high-mobility group box DNA-binding protein 1 (WDHD1), also recognized as acidic nucleoplasmic DNA-binding protein 1 (AND-1), and human chromosome transmission fidelity factor 4 (CTF4), is a relatively evolutionarily conserved protein with 1129 amino acids and homologs in most eukaryotes [56]. A recent investigation showed WDHD1 to modulate the cancer cell cycle checkpoint, engage in oncogene-induced re-replication, and impact tumor growth and metastasis [57]. Cui et al. discovered WDHD1 mRNA levels exhibited a substantial increase in more than 20 types of tumor tissues. Heightened WDHD1 expression is correlated with significantly shorter OS in ten tumors. Furthermore, WDHD1 expression showed a significant association with higher histological grades and pathological stages in uterine corpus endometrial carcinoma (UCEC) and liver hepatocellular carcinoma (LIHC) [58]. There is limited research on the aforementioned genes in HCC and this investigation has validated the effectiveness of the prognostic risk model, utilizing these five genes for precisely predicting the survival outcomes of HCC patients. This model enables a more personalized prognosis assessment and considers genetic variations and molecular differences among individual patients. It helps physicians better evaluate the survival risks of patients and formulate personalized treatment plans. By controlling the degradation or repressors of tumor promoters, E3 ligases play an important role in HCC, as evidence suggests. It has also been conclusively demonstrated that several E3 ligase targets regulate several significant HCC-related signal pathways and pathological processes, including the Wnt/-catenin pathway, PI3K/AKT/mTOR pathway, RAS/RAF/MEK/ERK pathway, and HBV infection [17]. The aforementioned findings correspond with the results of this study. However, the regulatory link between the E3 ubiquitin ligase-related ceRNA network remains unknown. E3 ubiquitin ligase-related ceRNA network regulation mechanism must be studied experimentally to find novel approaches for the personalized treatment of HCC.

Nomograms have recently been extensively applied as reliable and personalized cancer prognosis tools. Zhang et al. developed a nomogram that incorporated apoptotic-related lncRNA signature, age, stage, and T stage, and have demonstrated its clinical utility [59]. Many researchers have used ceRNA regulatory networks for studying molecular biological regulatory mechanisms that influence HCC patient prognosis [60]. Bai et al. developed an lncRNA predictive model for HCC patients based on 13 lncRNAs discovered via a ceRNA regulation network [61]. One investigation of ceRNA network identified potential biomarkers for the prediction of the recurrence of liver cancer [62]. circRNA, miRNA, and mRNA expression data and clinical information of HCC patients were extracted from the GEO, TCGA, and ICGC databases. Subsequently, a differential circRNA–miRNA–mRNA regulation network was built and essential genes were identified and confirmed. By combining this network with the online tool-based ceRNA network, an HCC-specific ceRNA network was generated and regulating circRNA–miRNA–mRNA axes were obtained. The CRE3UL signature was combined with clinicopathologic characteristics to predict prognosis using a nomogram.

Personalized therapeutic strategies for HCC still remain a significant challenge [63]. In this research, a prognostic signature using 5-CRE3UL in HCC was suggested and its validity was confirmed in two independent datasets on distinct platforms. The prognostic immune signature can further classify patients defined by clinical criteria [for example, early stage (I and II) and late stage (III and IV)] into subgroups with varying survival outcomes. Univariate COX analysis found there to be a significant correlation between TNM stage and the CRE3UL signature with HCC prognosis. Multivariate COX analysis showed that both TNM stage and the prognostic signature could serve as independent prognostic factors. As a consequence, the prognostic signature in this study has potential for personalized prognosis, diagnosis, and treatment of HCC patients and can be readily applied in clinical practice. In conclusion, the proposed CRE3UL signature offers precise prognostic predictions for HCC patients and provides valuable guidance for the formulation of specific treatment strategies by clinicians. In addition, it elucidates the association between this signature and the responsiveness to immune checkpoints and targeted medications. These findings could enhance the efficacy of therapeutic interventions for HCC patients [64]. Despite promising predictive performance in research, there may be obstacles to transitioning gene signatures into routine clinical practice, including the acceptance of new technologies by healthcare professionals, patient understanding and acceptance, and the challenges of formulating corresponding treatment decisions. In conclusion, further validation, standardization, and the overcoming of implementation barriers will be necessary before the integration of these findings into everyday clinical practice will be possible.

Although many databases were utilized to create the CRE3UL signature, there are still deficiencies. First, despite the validation in two cohorts, notable heterogeneity persisted among tumor samples marked by diverse regional and ethnic attributes, and even within individual samples. Previous investigations have found tumor heterogeneity to markedly impact the effectiveness of immunotherapy or chemotherapy. Second, the research sample was taken from a retrospective investigation and the results must be confirmed by a prospective multicenter study with a bigger sample size. Therefore, conducting extensive prospective studies and undertaking functional and mechanistic experiments to validate and elucidate the connection between E3 ubiquitin ligase and HCC are essential. Third, the HCC data and samples that were utilized in this study were sourced from the TCGA and ICGC databases. Detailed information on the specific etiology, such as distinguishing between viral and non-viral liver diseases, was regrettably not available in the publicly accessible datasets that were used in this study. While the significance of this aspect is recognized, the limitation lies in the inherent constraints of the TCGA and ICGC databases, in which comprehensive clinical information, including detailed liver disease etiology, may not be uniformly provided. The provided clinical information is also not sufficient for the application of the Barcelona Clinic Liver Cancer (BCLC) staging system. In future studies, every effort must be made to incorporate detailed information on the etiology of liver disease, particularly distinguishing between viral and non-viral origins if it is available in the datasets or by collaborating with institutions that possess such detailed clinical data. Furthermore, the clinical information for the application of the BCLC staging system must be enhanced. Finally, this study utilized LASSO multivariate Cox regression analysis to establish the model, but his method has certain limitations, including excessive penalization, selective bias, parameter estimation bias, and restriction to linear relationships. In contrast, machine learning models have greater representational power, which allows for nonlinear relationships to be captured and reduces overfitting risks through ensemble learning methods. Therefore, machine learning [65] should be employed to further enhance the methodology in subsequent research.

## Conclusions

A CRE3UL signature was developed for predicting the prognosis of HCC patients, which may assist doctors in making treatment choices. Furthermore, the nomogram that combined clinical characteristics and the CRE3UL signature was superior to TNM staging in clinical prognosis. It is anticipated that the nomogram will offer new insights into prognostic prediction and therapy for HCC patients. This provides valuable guidance for clinicians in devising specific personalized treatment strategies.

### Supplementary Information

Additional file 1.

Additional file 2.

Additional file 3.

Additional file 4.

Additional file 5.

Additional file 6.

Additional file 7.

Additional file 8.

## Data Availability

GSE97332 and GSE94508 datasets were soured from the GEO (https://www.ncbi.nlm.nih.gov/geo/) database.
