# Supplementary material for: Establishment of a circRNA-regulated E3 ubiquitin ligase signature and nomogram to predict immunotherapeutic efficacy and prognosis in hepatocellular carcinoma
Source: Eur J Med Res. 2024 Jun 10;29:318. doi: 10.1186/s40001-024-01893-6 (PMC11163726; doi:10.1186/s40001-024-01893-6)
Supplement: Supplementary file 1 — Additional file 1. [file 40001_2024_1893_MOESM1_ESM.docx]

**Figure S1.** Overview of UBcluster-related immune infiltration. **(A)** The box plot presents the relative composition of multiple cell types in the UB cluster A and B. **(B)** GSVA of biological pathways between the two distinct clusters. **(C)** The violin plot shows the difference in the ESTIMATE, Immune, and Stromal Scores between the two clusters. **(D)** Comparison of immune function in the UB cluster A and B. **(E)** Differences in the expression levels of immune checkpoint proteins between the UB cluster A and B. *, **, ***, and **** indicate a significance level of 0.05, 0.01, 0.001, and 0.0001, respectively.

**Figure S2.** Prognostic value of the risk model of five E3 ubiquitin ligases. **(A, B)** Risk score distribution, survival status, and the expression of five CRE3UL signature genes for patients in the low- and high-risk groups from four datasets (TCGA training set, TCGA validation set, whole TCGA cohort, and ICGC cohort). **(C)** Kaplan–Meier survival plots of the low- and high-risk groups from four datasets for OS. **(D)** tROC curves of the model formed by five E3 ubiquitin ligases in the four datasets.

**Figure S3.** Subgroup analyses of the overall survival of HCC patients in the TCGA database. **(A)** Age ≤ 65 years. **(B)** Age > 65 years. **(C)** Female. **(D)** Male. **(E)** G1+2. **(F)** G3+4 **(G)** Stage I+II. **(H)** Stage III+IV.

**Figure S4.** Comparing the prognostic precision of the model and clinical features and comparing risk scores between subgroups with different clinical characteristics. (A) Prognostic accuracy of the risk scores, age, gender, grade, and tumour stage were compared using time-dependent ROC curves in the time duration of 1 year. (B) The concordance index was used for comparing the discrimination of the model, age, gender, grade, and tumour stage in TCGA datasets. Risk scores are grouped by different clinical characteristics in the entire set. (C) Grade. (D) Stage. (E) Gender. (F) Age.

**Figure S5.** Principal component analysis between the high-risk and low-risk groups based on **(A)** TCGA genome expression profile of five E3 ubiquitin ligases, **(B)** ICGC genome expression profile of five E3 ubiquitin ligases. t-SNE2 analysis between the high-risk and low-risk groups based on **(C)** TCGA genome expression profile of five E3 ubiquitin ligases, **(D)** ICGC genome expression profile of five E3 ubiquitin ligases.

**Figure S6.** The therapeutic benefit of risk score. **(A–D)** Correlation between the CRE3UL signature and IC50 values of chemotherapy and targeted drugs, including **(A)** gefitinib, **(B)** nilotinib, **(C)** gemcitabine, and **(D)** sorafenib. **(E)** The relative distribution of dysfunction was compared between the low- and high-risk groups. **(F)** The relative distribution of exclusion was compared between the low- and high-risk groups. **(G)** The relative distribution of tide was compared between the low- and high-risk groups. **(H)** The relative distribution of MSI Expr Sig was compared between the low- and high-risk groups. **(I)** Differences in the expression levels of immune checkpoint proteins between the HRG and LRG (***p < 0.001, **p < 0.01, *p < 0.05). The IPS in low- and high-risk groups. **(J–M)** [IPS-CTLA4-neg-PD1-neg **(J)**, IPS-CTLA4-neg-PD1-pos **(K)**, IPS-CTLA4-pos-PD1-pos **(L)**, IPS-CTLA4-pos-PD1-neg **(M**)].

**Table S1.** Clinical features of 342 patients with hepatocellular carcinoma.
