# Supplementary figures and images for: Establishment of a circRNA-regulated E3 ubiquitin ligase signature and nomogram to predict immunotherapeutic efficacy and prognosis in hepatocellular carcinoma
Source: Eur J Med Res. 2024 Jun 10;29:318. doi: 10.1186/s40001-024-01893-6 (PMC11163726; doi:10.1186/s40001-024-01893-6)

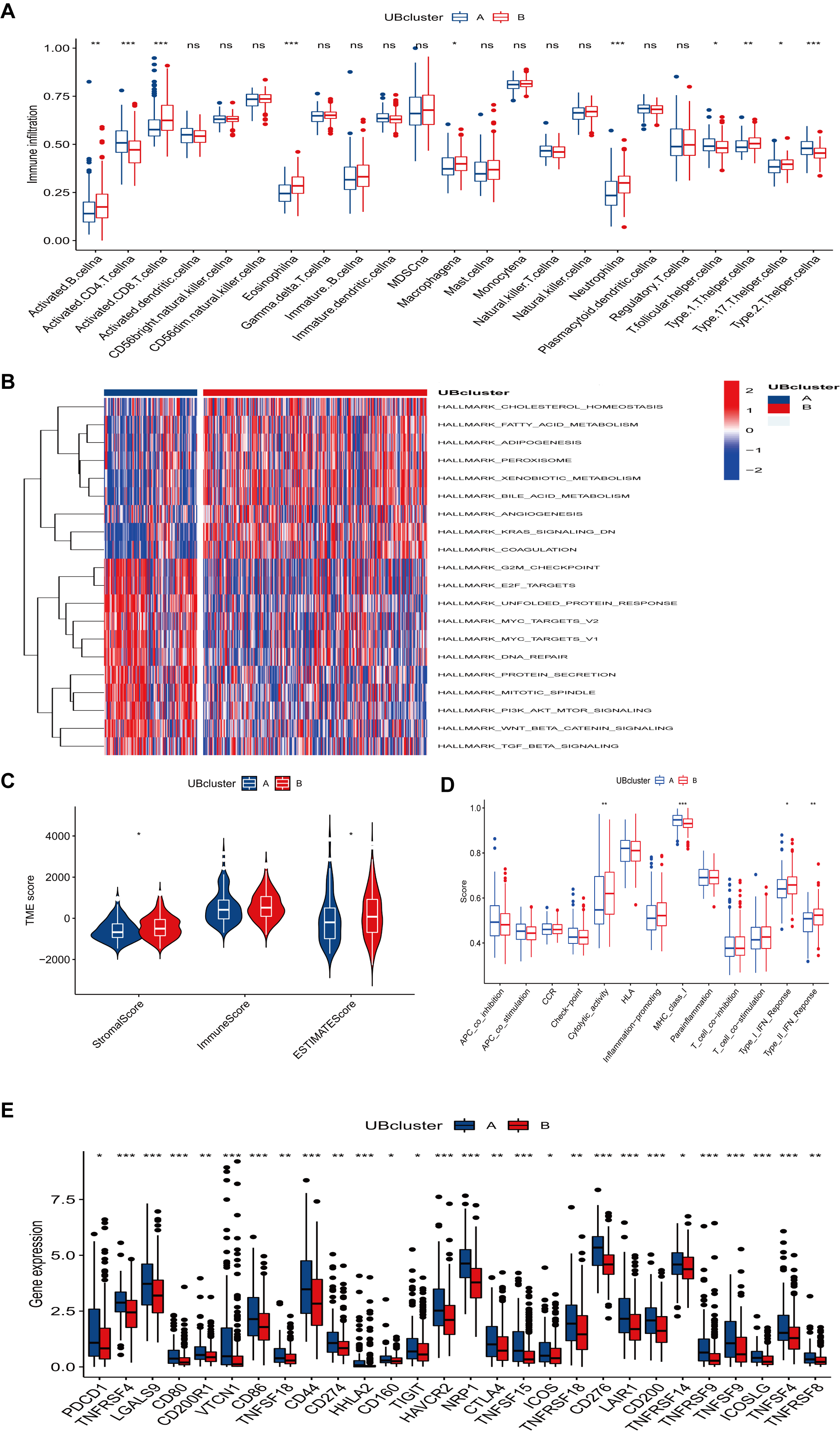

Supplement: Supplementary file 2 — Additional file 2. [file 40001_2024_1893_MOESM2_ESM.tif]

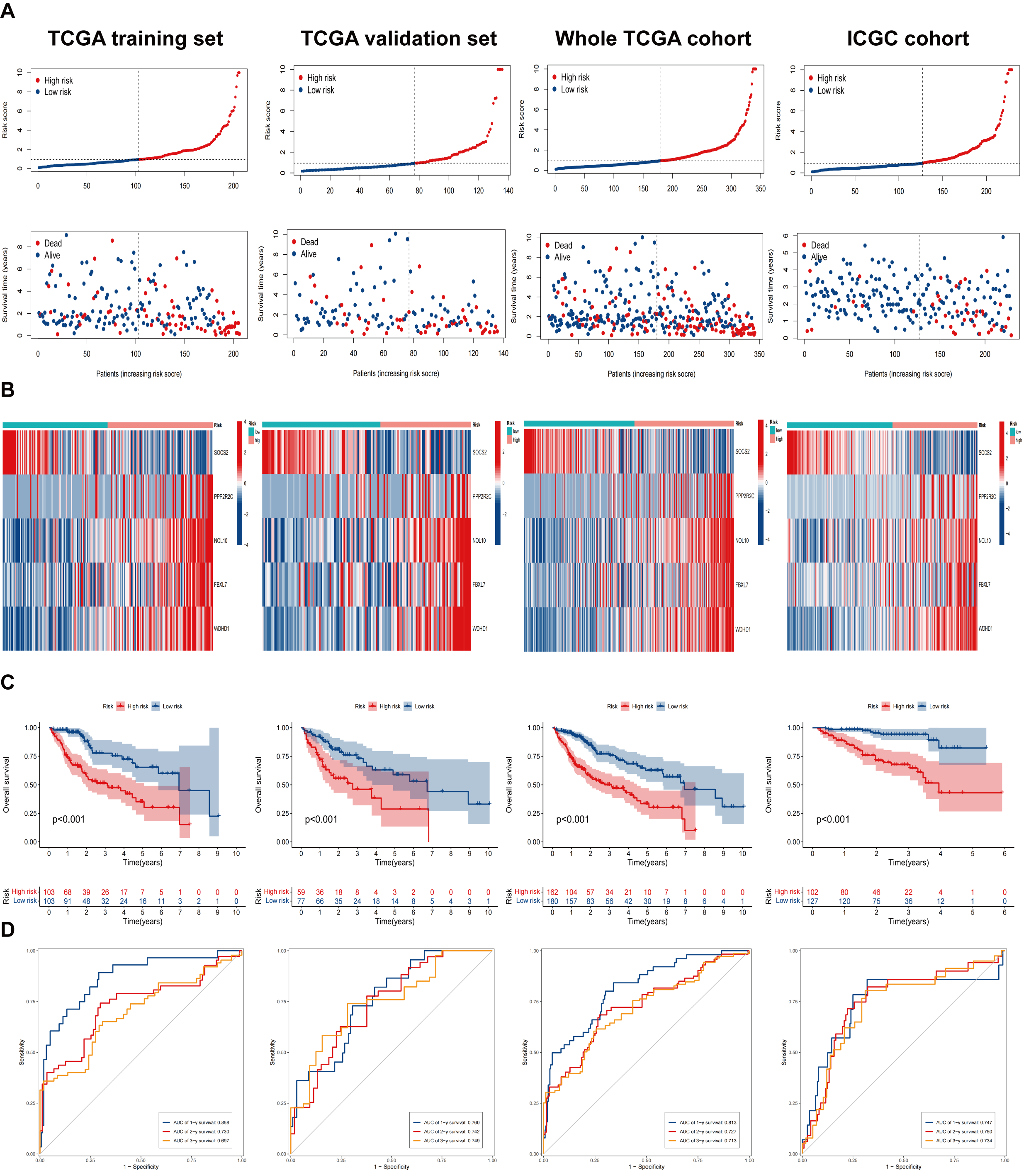

Supplement: Supplementary file 3 — Additional file 3. [file 40001_2024_1893_MOESM3_ESM.tif]

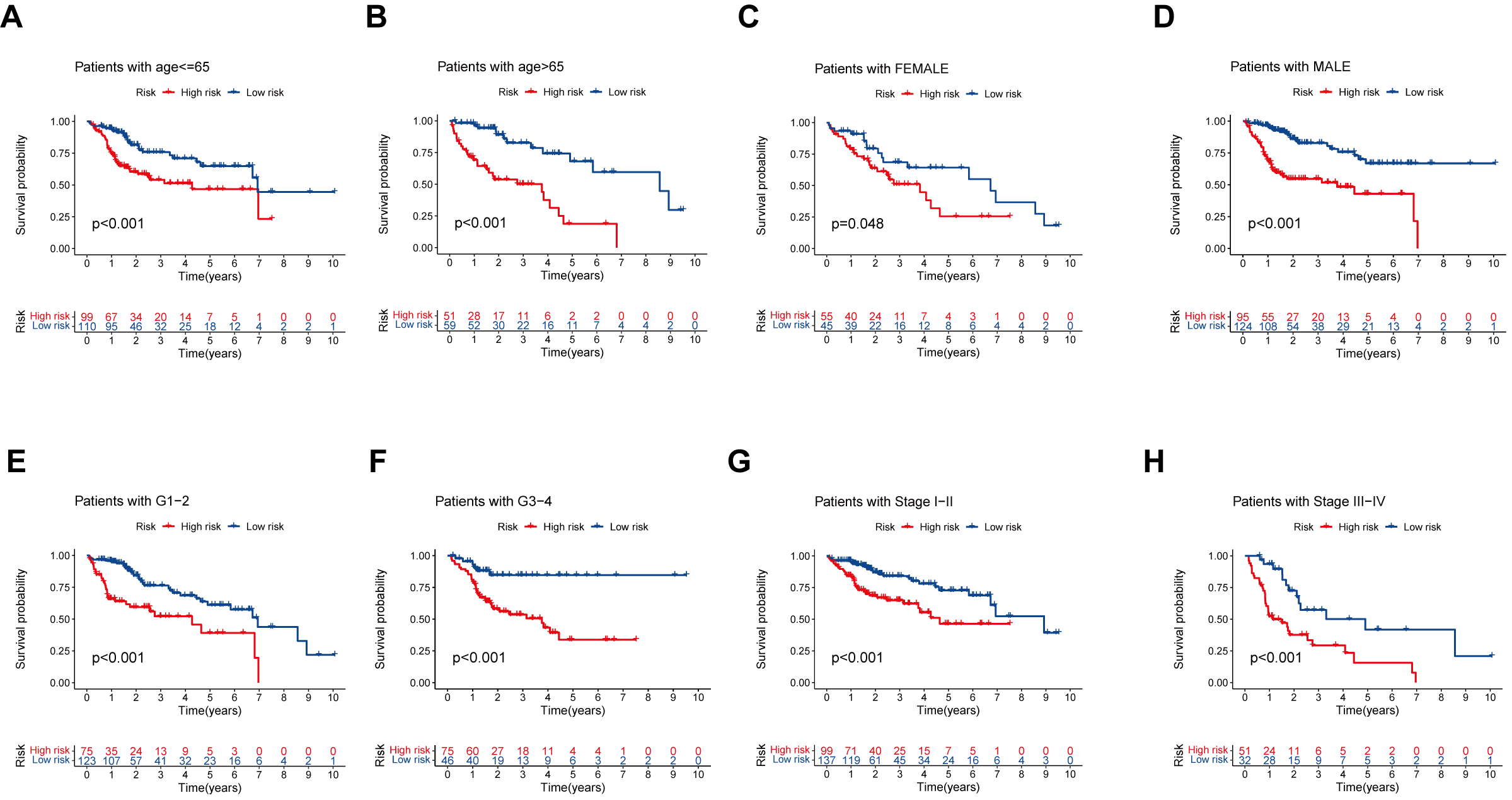

Supplement: Supplementary file 4 — Additional file 4. [file 40001_2024_1893_MOESM4_ESM.tif]

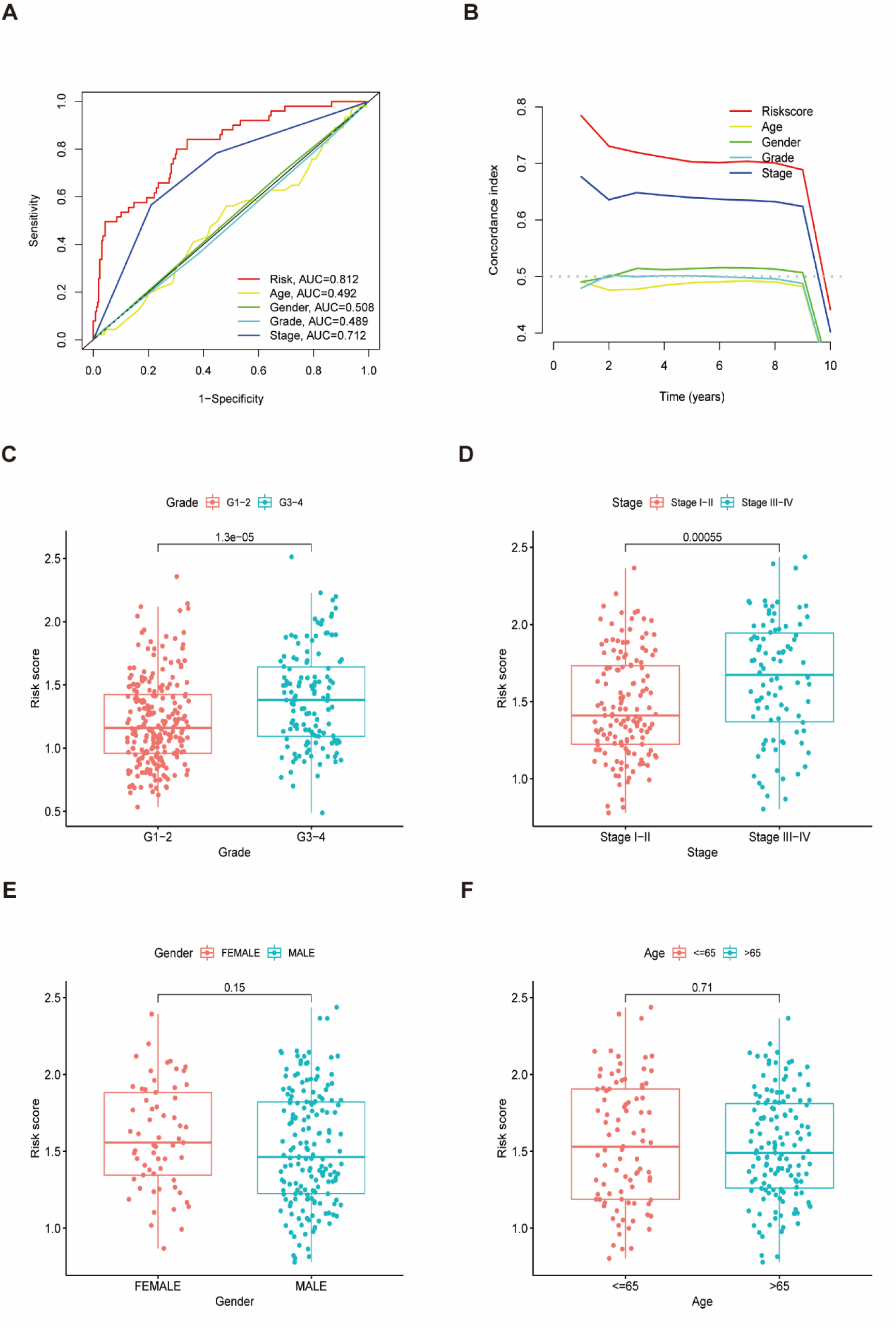

Supplement: Supplementary file 5 — Additional file 5. [file 40001_2024_1893_MOESM5_ESM.tif]

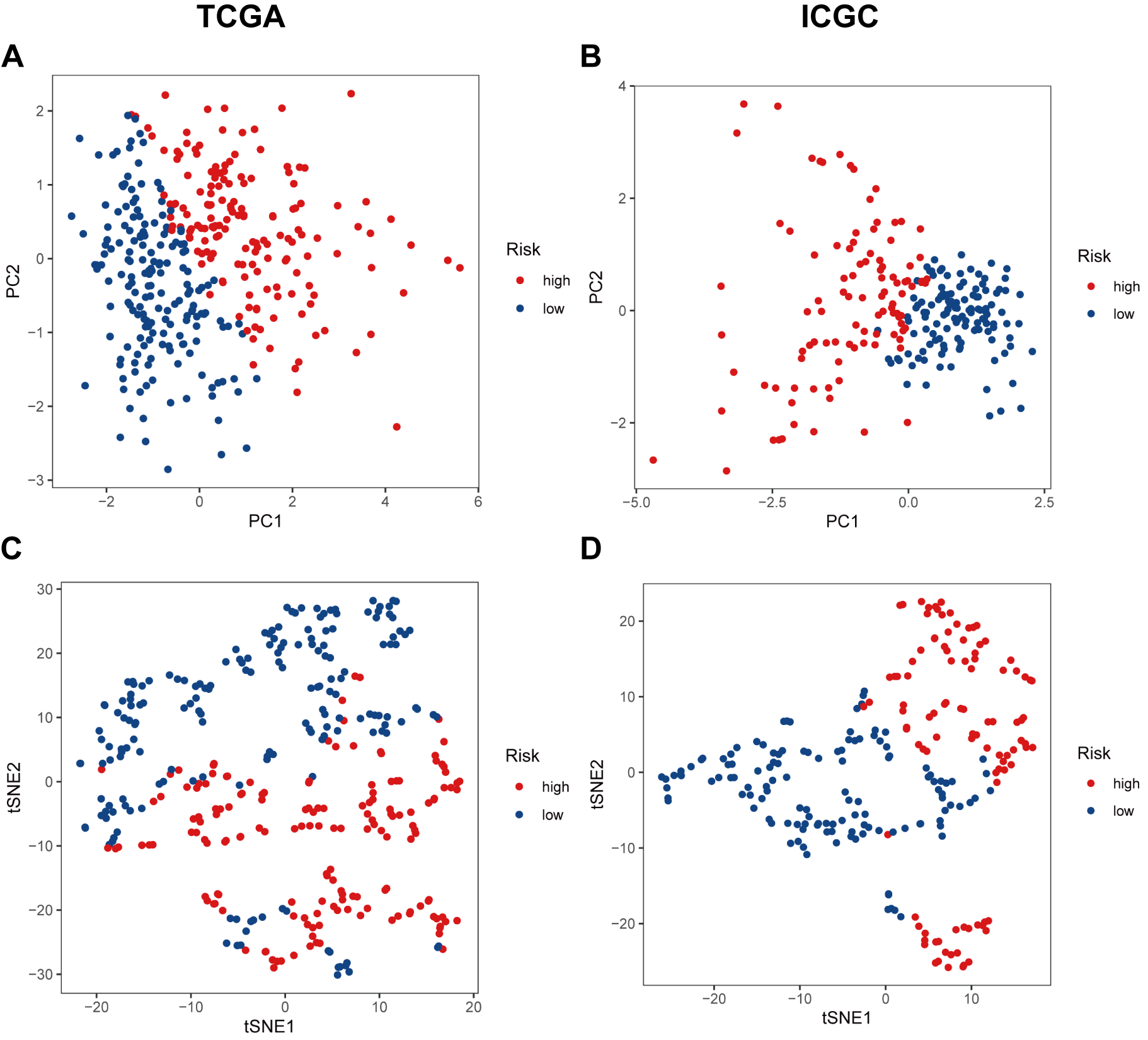

Supplement: Supplementary file 6 — Additional file 6. [file 40001_2024_1893_MOESM6_ESM.tif]

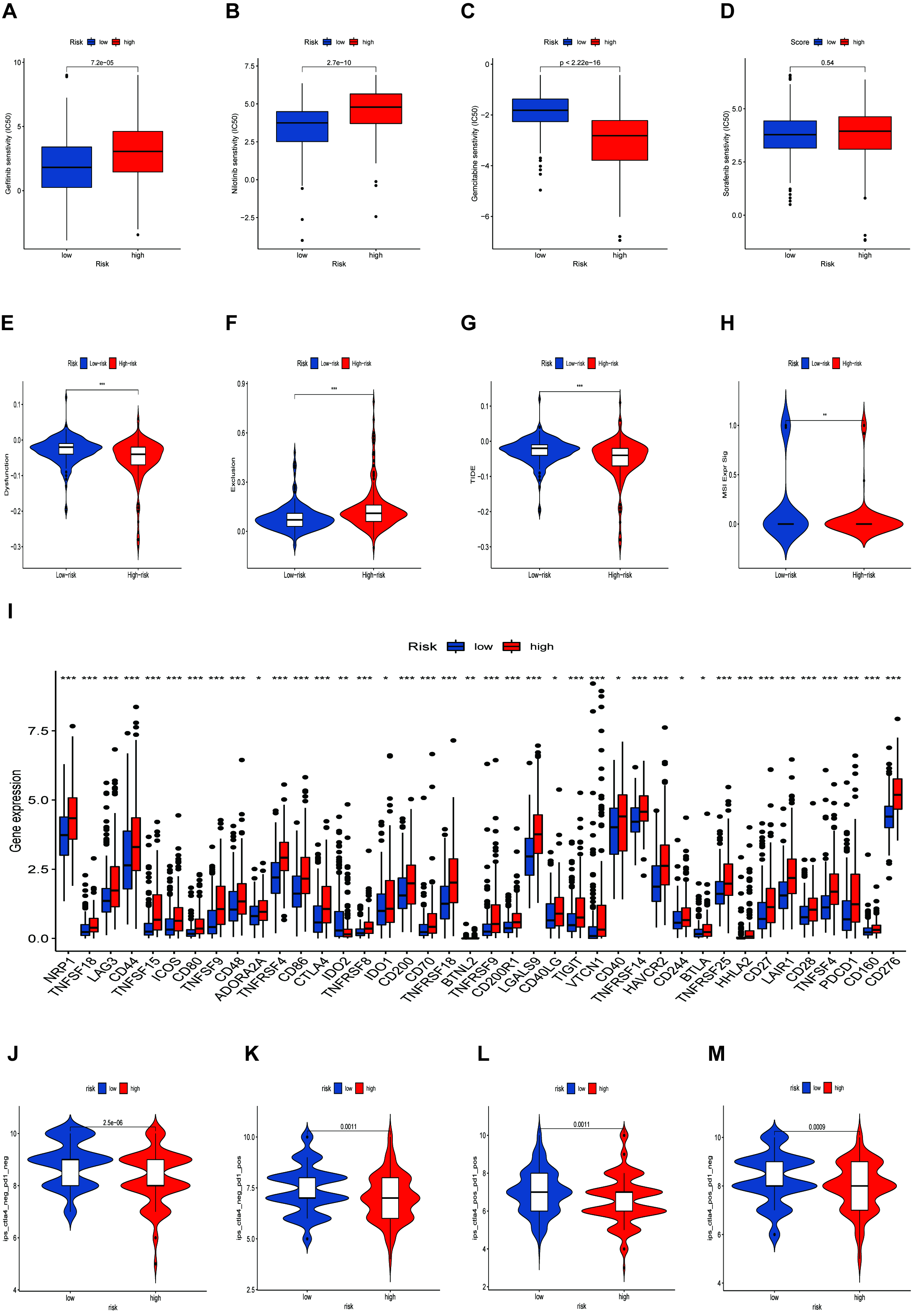

Supplement: Supplementary file 7 — Additional file 7. [file 40001_2024_1893_MOESM7_ESM.tif]
