# Supplementary material for: Establishment of a circRNA-regulated E3 ubiquitin ligase signature and nomogram to predict immunotherapeutic efficacy and prognosis in hepatocellular carcinoma
Source: Eur J Med Res. 2024 Jun 10;29:318. doi: 10.1186/s40001-024-01893-6 (PMC11163726; doi:10.1186/s40001-024-01893-6)
Supplement: Supplementary file 8 — Additional file 8. [file 40001_2024_1893_MOESM8_ESM.docx]

**Table1**

Clinical features of 342 patients with hepatocellular carcinoma.

| **Character** | **Training** **dataset** **n** **=** **206** | **Testing** **dataset** **n** **=** **136** | **Entire** **dataset** **n** **=** **342** | **P** **-value** |
| --- | --- | --- | --- | --- |
| **Age** |  |  |  | 0.8897 |
| ≤65 | 129 | 87 | 216 |  |
| >65 | 77 | 49 | 126 |  |
| **Gender** |  |  |  | 0.2506 |
| Female | 71 | 38 | 109 |  |
| Male | 135 | 98 | 233 |  |
| **Grade** |  |  |  | 0.8292 |
| G1–G2 | 126 | 88 | 214 |  |
| G3–G4 | 78 | 45 | 123 |  |
| Unknown | 2 | 3 | 5 |  |
| **TNM** **stage** |  |  |  | 0.5068 |
| I– II | 141 | 97 | 238 |  |
| III– IV | 52 | 31 | 83 |  |
| Unknown | 13 | 8 | 21 |  |
| **Tumour** **stage** |  |  |  | 0.2917 |
| T1–T2 | 148 | 104 | 252 |  |
| T3–T4 | 57 | 30 | 87 |  |
| Unknown | 1 | 2 | 3 |  |
